# Integrative genomic analysis of methylphenidate response in attention-deficit/hyperactivity disorder

**DOI:** 10.1038/s41598-018-20194-7

**Published:** 2018-01-30

**Authors:** Mireia Pagerols, Vanesa Richarte, Cristina Sánchez-Mora, Paula Rovira, María Soler Artigas, Iris Garcia-Martínez, Eva Calvo-Sánchez, Montse Corrales, Bruna Santos da Silva, Nina Roth Mota, Marcelo Moraes Victor, Luis Augusto Rohde, Eugenio Horacio Grevet, Claiton Henrique Dotto Bau, Bru Cormand, Miguel Casas, Josep Antoni Ramos-Quiroga, Marta Ribasés

**Affiliations:** 1 Psychiatric Genetics Unit, Group of Psychiatry, Mental Health and Addiction, Vall d’Hebron Research Institute (VHIR), Universitat Autònoma de Barcelona, Barcelona, Spain; 2 Department of Psychiatry, Hospital Universitari Vall d’Hebron, Barcelona, Spain; 3 Biomedical Network Research Centre on Mental Health (CIBERSAM), Instituto de Salud Carlos III, Barcelona, Spain; 4 Department of Psychiatry and Legal Medicine, Universitat Autònoma de Barcelona, Barcelona, Spain; 5 Department of Genetics, Institute of Biosciences, Universidade Federal do Rio Grande do Sul, Porto Alegre, Brazil; 6 Department of Human Genetics and Psychiatry, Donders Institute for Brain, Cognition and Behaviour, Radboud University Medical Centre, Nijmegen, The Netherlands; 7 ADHD Outpatient Program, Adult Division, Hospital de Clínicas de Porto Alegre, Porto Alegre, Brazil; 8 Department of Psychiatry, Faculty of Medicine, Universidade Federal do Rio Grande do Sul, Porto Alegre, Brazil; 9 Departament de Genètica, Microbiologia i Estadística, Facultat de Biologia, Universitat de Barcelona, Barcelona, Spain; 10 Centro de Investigación Biomédica en Red de Enfermedades Raras (CIBERER), Instituto de Salud Carlos III, Barcelona, Spain; 11 Institut de Biomedicina de la Universitat de Barcelona (IBUB), Barcelona, Spain; 12 Institut de Recerca Sant Joan de Déu (IR-SJD), Esplugues de Llobregat, Spain

## Abstract

Methylphenidate (MPH) is the most frequently used pharmacological treatment in children with attention-deficit/hyperactivity disorder (ADHD). However, a considerable interindividual variability exists in clinical outcome. Thus, we performed a genome-wide association study of MPH efficacy in 173 ADHD paediatric patients. Although no variant reached genome-wide significance, the set of genes containing single-nucleotide polymorphisms (SNPs) nominally associated with MPH response (P < 0.05) was significantly enriched for candidates previously studied in ADHD or treatment outcome. We prioritised the nominally significant SNPs by functional annotation and expression quantitative trait loci (eQTL) analysis in human brain, and we identified 33 SNPs tagging *cis*-eQTL in 32 different loci (referred to as eSNPs and eGenes, respectively). Pathway enrichment analyses revealed an over-representation of genes involved in nervous system development and function among the eGenes. Categories related to neurological diseases, psychological disorders and behaviour were also significantly enriched. We subsequently meta-analysed the association with clinical outcome for the 33 eSNPs across the discovery sample and an independent cohort of 189 ADHD adult patients (target sample) and we detected 15 suggestive signals. Following this comprehensive strategy, our results provide a better understanding of the molecular mechanisms implicated in MPH treatment effects and suggest promising candidates that may encourage future studies.

## Introduction

Attention-deficit/hyperactivity disorder (ADHD) is a neurodevelopmental disorder characterised by persistent and age-inappropriate symptoms of inattention, hyperactivity and/or impulsivity^1^, which significantly impacts on academic, social, emotional and psychological functioning. With a worldwide prevalence ranging from 5.3 to 7.1% in school-age children and adolescents^2^, ADHD is one of the most common childhood psychiatric conditions and causes high costs to the healthcare system and society^3,4^. Although its aetiology is largely unknown, several family, twin and adoption studies reported heritability estimates around 76%^5^, suggesting a strong genetic component in the pathogenesis of the disorder.

Among the wide variety of pharmacological options available in ADHD treatment, methylphenidate (MPH) is the first-line choice in paediatric patients, given its proved general efficacy in reducing ADHD symptoms and improving neuropsychological performance on executive functions^6,7^. However, a considerable interindividual variability exists in clinical outcome, optimal dosage and duration of effect^8,9^, which may reflect underlying genetic influences.

Most of the pharmacogenetic studies conducted so far in ADHD patients have focused on genes related to the catecholamine neurotransmission, with *SLC6A3* and *DRD4* being the most extensively investigated, since MPH is thought to exert its therapeutic effects through the inhibition of the dopamine and the norepinephrine transporters^10^. Based on this putative mechanism of action, additional genes such as *DRD2*, *DRD5, COMT*, *SLC6A2*, *ADRA2A, TPH2*, *SLC6A4*, *HTR1B*, *HTR2A* and *MAOA*^11^ have been considered plausible candidates that may influence medication response. Nevertheless, a recent review on ADHD pharmacogenetics in childhood reported no consistent effects for dopaminergic and serotoninergic signaling, and suggested neurodevelopmental genes as new promising targets^12^.

Given that candidate gene-based investigations have not reached many compelling results, genome-wide association studies (GWAS) may represent an alternative, hypothesis-free approach to unravel the molecular mechanisms implicated in MPH treatment. To date, only one prior GWAS evaluated the efficacy of a MPH transdermal system in 187 children with ADHD^13^. Although no genome-wide significant associations were found, the metabotropic glutamate receptor 7 (GRM7) and two SNPs within the *SLC6A2* gene showed potential involvement in MPH response. Using that sample, Mick *et al*.^14^ conducted a secondary GWAS of changes in blood pressure after MPH therapy and detected nominal evidence for genes functionally related to blood pressure regulation and other cardiovascular phenotypes, including a SNP in a K^+^-dependent Na^+^/Ca^2+^ exchanger (SLC24A3). Furthermore, despite the fact that GWAS have been useful to identify genetic risk loci for multiple complex conditions, yet the functional effects of the trait-associated variants and the underlying pathological mechanisms remain mainly elusive.

Based on the absence of clear conclusions regarding MPH response raised by previous genetic studies, we undertook a GWAS of MPH efficacy in 173 ADHD paediatric patients and, for the first time to our knowledge, we integrated data from functional annotation, expression quantitative trait loci (eQTL) and enrichment analyses to characterise the biological pathways associated with treatment response. Additionally, we performed a polygenic risk score analysis and a meta-analysis across the study sample and an independent population of 189 ADHD adult patients.

## Materials and Methods

### Discovery population

The study sample included 173 ADHD paediatric patients for whom MPH was prescribed. Subjects were required to satisfy full DSM-IV criteria for ADHD, be under 18 years of age, Spanish of Caucasian origin and have never received MPH treatment. Patients with an IQ below 70 or having pervasive developmental disorders were not eligible for the investigation. Additional exclusion criteria included schizophrenia or other psychotic disorders; adoption; sexual or physical abuse; birth weight <1.5 kg; any significant neurological or systemic disease that might explain ADHD symptoms; and clinical contra-indication to MPH. Comorbid oppositional defiant disorder, conduct disorder, depression and anxiety disorders were allowed unless determined to be the primary cause of ADHD symptomatology. The study was approved by the Ethics Committee of the Hospital Universitari Vall d’Hebron and all methods were performed in accordance with the relevant guidelines and regulations. Written informed consent was obtained from parents/caregivers.

### Clinical assessment

Diagnoses of ADHD and comorbidities were established by child psychiatrists blind to patients’ genotypes through the Present and Lifetime version of the Kiddie Schedule for Affective Disorders and Schizophrenia for School-Age Children (K-SADS-PL). Furthermore, families were interviewed with the Clinical Global Impression-Severity scale (CGI-S). Additional information on clinical assessment is available elsewhere^15^.

### Pharmacological intervention

Patients were treated according to the program’s recommendations of initiating treatment with MPH at low to moderate dose and titrating to higher doses until no further clinical improvement or limiting adverse effects were observed. The mean daily dose of MPH prescribed was 1.06 mg/kg (SD = 0.28). Risperidone was the most frequent concomitant drug.

### Treatment outcome

We considered the Clinical Global Impression-Improvement scale (CGI-I)^16^, which ranges from 1 (‘very much improved’) to 7 (‘very much worse’), as the primary outcome measure of treatment success. Those patients rated with a CGI-I score of two points or less after eight weeks of treatment were considered as responders, while the remaining were classified as non-responders.

### Genome-wide association study

Genomic DNA was isolated from peripheral blood leukocytes by a salting out procedure^17^. A total of 173 samples were genotyped on the Infinium PsychArray-24 BeadChip platform (Illumina, San Diego, CA, USA), which covers 588,628 markers, and processed at the Stanley Center for Psychiatric Research, Broad Institute of MIT and Harvard (Cambridge, MA, USA). Pre-imputation quality control and principal components analysis were implemented following the QC and PCA modules from the Ricopili with the default settings (https://sites.google.com/a/broadinstitute.org/ricopili/). Genotype imputation was performed with the pre-phasing and imputation strategy using the EUR population of the 1,000 Genomes Project Phase 1 dataset as the reference panel (http://www.1000genomes.org/). We assured the accuracy of the imputation data by filtering best-guess genotypes for an info score <0.3. This resulted in a total of 11,051,824 markers eligible for association tests.

Before GWAS analysis, further quality control measures were applied using the PLINK software^18^. Individuals exhibiting high rates of genotype missingness (>98%) were removed, as well as SNPs with low call rate (<0.99), MAF < 0.01 or failing Hardy-Weinberg equilibrium test (P < 1e-06).

Finally, 173 subjects and 3,566,199 variants were tested for association with MPH response through logistic regression under an additive model, which included those clinical variables (i.e., CGI-S baseline scores) and principal components (i.e., PC6) significantly associated with clinical outcome (P ≤ 0.05) as covariates.

### Identification of candidate causal SNPs

Among the SNPs showing nominal association with treatment outcome (P < 0.05), we used the genome pipeline of SNPinfo (http://snpinfo.niehs.nih.gov/)^19^ to prioritise those that were more likely to affect protein sequence, transcriptional regulation, mRNA splicing or miRNA binding based on functional annotation. GenomePipe parameter values included: GWAS population = CEU; study population = CEU; flanking region = 200,000 bp; GWAS P-value < 0.05; LD threshold = 0.8; and MAF = 0.01 for all prediction methods. Additionally, we combined both the predicted conserved transcription factor-binding sites (TFBS) with the regulatory potential score (RP Score; available at http://genome.ucsc.edu) to improve predictions as suggested in several studies^20–22^.

### *Cis*-expression quantitative trait loci analysis

*Cis*-eQTL analysis was conducted on 193 neuropathologically normal cortical samples of adult humans from Myers *et al*.^23^. Expression-genotype pairs were extracted after extending the genotyped data by imputation as previously described, and considering a 10 kb window around the untranslated regions. Rank-invariant normalised expression levels were log_10_ transformed and transcripts detected in less than 75% of the samples were discarded from the study. Association tests were performed under a linear model with the MatrixEQTL R Package^24^, including gender, age at death, cortical region, day of expression hybridisation, institute source of sample, post-mortem interval and transcripts detected rate in each sample as covariates.

### Functional and pathway enrichment analysis

The biological functions and pathways related to genes containing at least one SNP nominally associated with both MPH response and human cortical expression levels (referred to as eSNPs) were assessed through the Ingenuity Pathway Analysis software (IPA) (Ingenuity Systems, Redwood City, CA, USA; www.ingenuity.com). IPA was also used to test for over-representation of genes previously studied in either ADHD or treatment outcome. Candidate genes for ADHD or MPH response were selected based on the gene list provided by the ADHDgene database (http://adhd.psych.ac.cn/index.do)^25^ and a comprehensive search for published reviews of ADHD genetic and pharmacogenetic studies^11,12,26–31^. Thus, a total of 436 genes were considered (Supplementary Table S1). Fisher’s exact tests, with a Benjamini-Hochberg-adjusted P-value (P_B-H_) < 0.05 as significance threshold, were applied in all analyses. To achieve meaningful statistics and interpretation of the results, we restricted the enrichment analysis to those annotation terms that included ≥2 genes of our dataset.

### Polygenic risk score analysis

We generated polygenic risk scores (PRS) based on the results of the present GWAS using the Polygenic Risk Score software (PRSice)^32^. A logistic regression model was applied to test whether PRS at multiple stepwise P-value thresholds (i.e., P_T_ < 1e-04, P_T_ < 1e-03, P_T_ < 0.05, P_T_ < 0.1, P_T_ < 0.2, P_T_ < 0.3, P_T_ < 0.4, and P_T_ < 0.5) predicted treatment outcome in an independent sample of patients with ADHD (target population). The target population comprised 189 Brazilian adults from the Adult ADHD Outpatient Clinic of the Hospital de Clínicas de Porto Alegre, who underwent immediate-release MPH treatment. Diagnoses of ADHD and comorbidities, as well as inclusion/exclusion criteria, were achieved as previously described^33^. The outcome measures of MPH treatment were the CGI-S scale, applied before medication and at least four weeks after its beginning, and the CGI-I scale. Drug response was defined following the criteria used in the discovery sample. Similarly, samples were genotyped and imputed using the same platform, imputation protocol and reference panel. The resulting dataset consisted of 7,304,149 SNPs with an info score >0.6, a genotype call probability >0.8 and a missing rate <0.02.

Potential confounders were included as covariates in the PRS model if they were associated with MPH response (P ≤ 0.05) in the target sample (i.e., CGI-S baseline scores, use of concomitant medication and presence of phobia as comorbid condition), as well as the 10 first principal components to control for population stratification.

### Meta-analysis

The eSNPs nominally associated with MPH response in the discovery sample were meta-analysed across the discovery and the target population used in the PRS analysis by the inverse-variance weighted method. The threshold for significance was set at P ≤ 1.52e-03 under the more conservative Bonferroni correction, taking into account 33 SNPs.

### Data availability

The datasets generated and/or analysed during the current study are not publicly available due to ethics constraints but are available from the corresponding author on reasonable request.

## Results

### Genome-wide association study in the discovery population

Subjects were predominantly male (84.4%), with an average age at assessment of 9.59 (SD = 2.91) years (range 5–17). One hundred and thirty-one participants (75.7%) met DSM-IV criteria for ADHD-combined subtype, 37 (21.4%) had ADHD-inattentive subtype and 5 (2.9%) were diagnosed with ADHD-hyperactive-impulsive subtype. Comorbid disorders were present in a modest number of patients (22.5%), the main ones being disabilities in reading and writing (12.7%), oppositional defiant disorder (5.8%) and tic disorders (1.7%). One hundred and forty-one subjects (81.5%) responded favourably to treatment according to the CGI-I scale, while 32 (18.5%) failed to show a clinical response to MPH. Responders and non-responders were comparable with regard to age, sex, ADHD subtype, comorbidity, use of concomitant medication, MPH dose and drug formulation (P > 0.05). There were significant differences, however, in the severity of symptoms as assessed by the CGI-S scale (P < 1e-03), with children resistant to MPH scoring higher at the baseline evaluation than children showing clinical improvement (Supplementary Table S2).

No variant reached genome-wide significance (P < 5e-08). However, the set of 4,709 genes containing SNPs nominally associated with MPH response (P < 0.05; Supplementary Table S3) was significantly enriched for candidates previously studied in ADHD or treatment outcome, with 199 out of 436 being present in this category (ratio = 0.46; P_B-H_ = 1.56e-31).

### Identification of candidate causal SNPs and *cis*-expression quantitative trait loci analysis

Considering these results, we prioritised the SNPs with P-values below 0.05 based on functional annotation and eQTL analysis rather than focusing on the top significant hits. Eight hundred and ninety-six independent markers were selected as candidate causal variants by functional annotation (Supplementary Table S4) and were subjected to further *cis*-eQTL analysis on a pre-existing dataset of 193 neuropathologically normal human cortical samples^23^. After imputation and quality control, a total of 284 variants and 300 genes with detectable expression levels in at least 75% of the samples were available for 146 individuals. Of these, we identified 33 SNPs tagging *cis*-eQTL in 32 different loci (referred to as eGenes), with eight SNP-gene pairs surpassing the 0.05 false discovery rate (FDR) threshold: rs12302749-*SPSB2*, P_FDR_ = 1.13e-05; rs1061115-*PYROXD2*, P_FDR_ = 2.17e-04; rs2071421-*ARSA*, P_FDR_ = 7.26e-04; rs11553441-*RRP7A*, P_FDR_ = 7.26e-04; rs4902333-*CHURC1*, P_FDR_ = 7.26e-04; rs17279558-*GGH*, P_FDR_ = 0.013; rs9901673-*SENP3*, P_FDR_ = 0.023; and rs17685420-*PEBP4*, P_FDR_ = 0.041 (Table 1).

**Table 1:** *Cis*-associated gene-SNP pairs with a nominal significant effect on methylphenidate response in the GWAS analysis.

Gene	Chr^a^	Start base^b^	Stop base^c^	SNP	SNP base^d^	Risk allele	OR (95% CI)	GWAS P-value	Beta	eQTL P-value	eQTL adjusted P-value (FDR)^e^
*SPSB2*	12	6870935	6873357	rs12302749	6867132	T	2.31 (1.22–4.39)	0.011	0.115	3.34e-08	1.13e-05
*PYROXD2*	10	98383565	98415221	rs1061115	98417292	G	2.23 (1.13–4.41)	0.021	−0.088	1.28e-06	2.17e-04
*ARSA*	22	50622754	50628173	rs2071421	50625988	T	2.56 (1.06–6.22)	0.037	0.104	8.26e-06	7.26e-04
*RRP7A*	22	42508335	42519823	rs11553441	42516091	C	3.13 (1.15–8.54)	0.026	0.177	1.04e-05	7.26e-04
*CHURC1*	14	64914361	64935368	rs4902333	64909368	T	2.37 (1.24–4.51)	8.73e-03	0.116	1.07e-05	7.26e-04
*GGH*	8	63015079	63039051	rs17279558	63015187	C	3.61 (1.12–11.7)	0.032	0.130	2.38e-04	0.013
*SENP3*	17	7561992	7571969	rs9901673	7580783	A	3.95 (1.79–8.71)	6.53e-04	0.052	4.66e-04	0.023
*PEBP4*	8	22713251	22941095	rs17685420	22927888	T	2.87 (1.38–5.94)	4.62e-03	−0.073	9.72e-04	0.041
*STRBP*	9	123109494	123268576	rs9032	123104493	C	2.28 (1.07–4.85)	0.033	0.071	2.15e-03	0.081
*ETFDH*	4	158672101	158708713	rs11559290	158680524	C	2.08 (1.01–4.28)	0.048	−0.048	2.57e-03	0.087
*CORO7*	16	4354542	4416961	rs3810818	4382028	A	2.10 (1.13–3.94)	0.020	−0.053	3.17e-03	0.098
*FXR2*	17	7591230	7614897	rs9901675	7581494	A	4.12 (1.32–12.9)	0.015	0.130	3.84e-03	0.107
*NFIB*	9	14081843	14398983	rs7858	14087770	C	2.89 (1.02–8.19)	0.045	−0.055	4.12e-03	0.107
*ALDH1L1*	3	126103561	126181526	rs2886059	126146923	C	2.73 (1.04–7.14)	0.041	−0.078	5.31e-03	0.129
*OPCML*	11	132403361	133532983	rs751655	132623600	C	3.08 (1.18–8.02)	0.022	−0.063	7.43e-03	0.158
*PURA*	5	140114123	140119416	rs2013169	140118020	T	3.32 (1.23–9.01)	0.018	0.071	7.45e-03	0.158
*ZDHHC7*	16	84974460	85011732	rs3210967	84975857	C	2.09 (1.11–3.94)	0.023	0.056	8.03e-03	0.160
*WRB*	21	39380287	39397889	rs3761372	39371919	T	3.38 (1.39–8.22)	7.36e-03	0.071	8.54e-03	0.161
*FARP2*	2	241356249	241494842	rs757978	241431686	C	5.18 (1.19–22.6)	0.029	0.062	0.010	0.181
*SENP3*	17	7561992	7571969	rs11552708	7559238	A	3.81 (1.50–9.72)	5.05e-03	0.041	0.011	0.189
*ZNF565*	19	36182060	36215084	rs4805162	36183403	G	2.24 (1.19–4.22)	0.012	0.034	0.016	0.255
*ESYT2*	7	158730998	158829628	rs1061735	158733764	G	2.91 (1.10–7.67)	0.031	0.030	0.017	0.255
*HTT*	4	3074510	3243960	rs362272	3233253	G	2.40 (1.06–5.42)	0.035	−0.033	0.019	0.276
*CMTM8*	3	32238679	32370325	rs4627790	32259860	C	1.98 (1.07–3.68)	0.030	0.054	0.021	0.298
*ZNF134*	19	57614219	57624717	rs10413455	57620255	A	5.75 (1.35–24.4)	0.018	0.061	0.024	0.323
*PDIA2*	16	283118	287209	rs1048786	286916	C	3.55 (1.19–10.6)	0.023	−0.087	0.027	0.345
*PIGM*	1	160027672	160031993	rs12409352	160030645	A	2.67 (1.00–7.12)	0.049	0.029	0.032	0.395
*TRIB3*	20	380629	397559	rs2295490	388261	G	2.06 (1.06–4.00)	0.033	0.092	0.034	0.406
*ZNF211*	19	57633167	57644046	rs10420097	57633193	G	7.29 (1.82–29.3)	5.18e-03	0.084	0.038	0.439
*ARHGAP12*	10	31805398	31928876	rs2799018	31913141	T	1.89 (1.00–3.56)	0.049	−0.036	0.039	0.446
*ARHGEF28*	5	73626158	73941993	rs929740	73621913	G	2.52 (1.31–4.84)	5.40e-03	−0.037	0.042	0.453
*CDH23*	10	71396934	71815947	rs17712523	71777857	G	2.61 (1.05–6.48)	0.039	−0.073	0.045	0.475
*ELP5*	17	7252053	7259940	rs4562	7260420	A	2.22 (1.24–3.95)	6.95e-03	−0.031	0.048	0.497

Note: SNP, single-nucleotide polymorphism; GWAS, genome-wide association study; Chr, gene chromosomal location; OR, odds ratio; CI, confidence interval; eQTL, expression quantitative trait loci.

^a,b,c,d^All relative to the human reference genome GRCh38 (NCBI Build 38).

^e^Significance threshold for the False Discovery Rate (FDR) correction at P < 0.05.

### Functional and pathway enrichment analysis

The set of 32 eGenes included three candidates previously investigated in ADHD, namely *ALDH1L1*^34^, *CDH23*^35^ and *CMTM8*^36^ (ratio = 0.007; P_B-H_ = 0.023), and showed over-representation of genes implicated in abnormal morphology of molecular layer of cerebellum (P_B-H_ = 0.012), abnormal morphology of white matter (P_B-H_ = 0.012), morphology of axons (P_B-H_ = 0.012), morphology and length of neurites (P_B-H_ = 0.012 and P_B-H_ = 0.021, respectively), coordination (P_B-H_ = 0.022), and formation of hippocampus (P_B-H_ = 0.033). Interestingly, categories related to neurological diseases, psychological disorders and behaviour were also significantly enriched, including learning deficit (P_B-H_ = 0.012), hyperactive behaviour (P_B-H_ = 0.015) and spatial learning (P_B-H_ = 0.018) (Table 2).

**Table 2:** Significantly enriched biological functions and diseases identified by Ingenuity Pathway Analysis within the eGenes associated with methylphenidate response.

Categories	Diseases or functions annotation	Adjusted P-value (Benjamini-Hochberg)^a^	Molecules
Nervous System Development and Function, Organ Morphology, Organismal Development	abnormal morphology of molecular layer of cerebellum	0.012	ARSA, PURA
Nervous System Development and Function, Organ Morphology, Tissue Morphology	abnormal morphology of white matter	0.012	ARSA, PURA
Cellular Development, Embryonic Development, Organismal Development	differentiation of neuronal progenitor cells	0.012	FXR2, HTT
Developmental Disorder, Neurological Disease	learning deficit	0.012	ARSA, HTT
Cell Morphology, Nervous System Development and Function, Organ Morphology, Organismal Development, Tissue Morphology	morphology of granule cells	0.012	HTT, NFIB
Cell Morphology, Haematological System Development and Function, Nervous System Development and Function	morphology of microglia	0.012	ARSA, HTT
Neurological Disease	gait disturbance	0.012	ARSA, HTT, PURA
Cell Morphology, Nervous System Development and Function, Tissue Morphology	morphology of axons	0.012	ARSA, HTT, PURA
Cell Morphology, Nervous System Development and Function	morphology of neuroglia	0.012	ARSA, HTT, NFIB
Cell Morphology, Nervous System Development and Function, Organ Morphology, Organismal Development	morphology of brain cells	0.012	ARSA, HTT, NFIB, PURA
Cell Morphology, Nervous System Development and Function, Tissue Morphology	morphology of neurites	0.012	ARSA, FARP2, HTT, PURA
Cell Morphology, Nervous System Development and Function, Tissue Morphology	morphology of neurons	0.012	ARSA, CDH23, FARP2, HTT, NFIB, PURA
Neurological Disease	late-onset encephalopathy	0.014	ARSA, HTT
Psychological Disorders	hyperactive behaviour	0.015	ARSA, FXR2, HTT
Neurological Disease	tremor	0.015	ARSA, HTT, PURA
Nervous System Development and Function, Organ Morphology, Organismal Development	abnormal morphology of dentate gyrus	0.015	NFIB, PURA
Cell Morphology, Nervous System Development and Function, Organ Morphology, Organismal Development, Tissue Morphology	abnormal morphology of Purkinje cells	0.015	ARSA, PURA
Cell Death and Survival, Cellular Compromise, Neurological Disease, Organismal Injury and Abnormalities, Tissue Morphology	neurodegeneration of Purkinje cells	0.015	ARSA, HTT
Nervous System Development and Function, Organ Morphology, Organismal Development	abnormal morphology of telencephalon	0.015	ARSA, HTT, NFIB
Behaviour	spatial learning	0.018	ARSA, FXR2, HTT
Nervous System Development and Function, Organ Morphology, Organismal Development	mass of brain	0.019	HTT, PURA
Cell Morphology, Cellular Function and Maintenance, Nervous System Development and Function, Tissue Morphology	length of neurites	0.021	FARP2, HTT
Organismal Injury and Abnormalities	abnormality of head	0.022	HTT, NFIB
Nervous System Development and Function	coordination	0.022	ARSA, FXR2, HTT
Cellular Development	differentiation of stem cells	0.022	FXR2, HTT, NFIB
Developmental Disorder, Neurological Disease, Organismal Injury and Abnormalities	cerebral dysgenesis	0.022	NFIB, PURA
Nervous System Development and Function, Organ Morphology, Organismal Development	morphology of cerebral cortex	0.023	HTT, NFIB, PURA
Organismal Development	size of head	0.024	HTT, NFIB, PURA
Neurological Disease, Organismal Injury and Abnormalities	astrocytosis	0.025	ARSA, HTT
Cell Death and Survival, Cellular Compromise, Neurological Disease, Tissue Morphology	neurodegeneration of axons	0.026	ARSA, HTT
Cellular Growth and Proliferation, Nervous System Development and Function, Organ Development	proliferation of brain cells	0.030	HTT, PURA
Nervous System Development and Function, Organ Morphology, Organismal Development	abnormal morphology of brain	0.030	ARSA, HTT, NFIB, PURA
Embryonic Development, Organismal Development, Tissue Development	mesoderm development	0.032	CHURC1, HTT
Embryonic Development, Nervous System Development and Function, Organ Development, Organismal Development, Tissue Development	formation of hippocampus	0.033	HTT, NFIB
Nervous System Development and Function, Organ Morphology, Tissue Morphology	quantity of brain cells	0.042	HTT, PURA
Nervous System Development and Function	sensation	0.047	CDH23, FXR2, HTT

Note: eGenes, genes whose expression levels are associated with at least one genetic variant.

^a^Significance threshold for the Benjamini-Hochberg correction at P < 0.05.

### Polygenic risk score analysis and meta-analysis using the target population

Finally, in order to assess the predictive value of our findings we first computed PRS derived from the present GWAS in an independent sample of ADHD adult patients for whom data on response to MPH were available. The demographic and clinical characteristics of the target population according to the response status are presented in Supplementary Table S5. Briefly, 85.2% of subjects (n = 161) were classified as responders, while 14.8% (n = 28) exhibited a reduced or lack of improvement. Responders and non-responders significantly differed with regard to CGI-S baseline scores, use of concomitant medication and presence of phobia as comorbid condition, and thus these additional risk factors were entered as covariates in the PRS model, as well as the 10 first principal components to control for population stratification. Since we did not detect significant results at any of the predefined P-value thresholds, we subsequently focused on the 33 eSNPs nominally associated with treatment outcome in the discovery sample and we increased statistical power by performing a meta-analysis across the discovery and the target population. Sixteen suggestive signals were identified (Table 3). Among them, 15 revealed the same direction of effect, with rs17685420 in the *PEBP4* gene being significant after Bonferroni correction (OR = 3.07 (1.76–5.35), P = 7.90e-05), followed by additional compelling markers such as rs2071421 within *ARSA* (OR = 2.63 (1.29–5.37), P = 7.71e-03), rs2886059 in *ALDH1L1* (OR = 2.30 (1.14–4.66), P = 0.020), and rs17712523 in *CDH23* (OR = 2.13 (1.07–4.24), P = 0.031).

**Table 3:** Meta-analysis of the eSNPs nominally associated with methylphenidate response across the discovery and the target population.

SNP	Chr^a^	SNP base^b^	Risk allele	SPAIN		BRAZIL		META-ANALYSIS		Gene
				OR (95% CI)	P-value	OR (95% CI)	P-value	OR (95% CI)	P-value^c^	
rs17685420	8	22927888	T	2.87 (1.38–5.94)	4.62e-03	3.38 (1.43–8.01)	5.71e-03	3.07 (1.76–5.35)	7.90e-05	*PEBP4*
rs3210967	16	84975857	C	2.09 (1.11–3.94)	0.023	2.25 (1.02–4.94)	0.044	2.15 (1.31–3.52)	2.40e-03	*ZDHHC7*
rs10413455	19	57620255	A	5.75 (1.35–24.4)	0.018	3.86 (0.63–23.7)	0.144	4.93 (1.59–15.3)	5.70e-03	*ZNF134*
rs2071421	22	50625988	T	2.56 (1.06–6.22)	0.037	2.76 (0.84–9.15)	0.096	2.63 (1.29–5.37)	7.71e-03	*ARSA*
rs12302749	12	6867132	T	2.31 (1.22–4.39)	0.011	1.49 (0.72–3.10)	0.280	1.91 (1.18–3.09)	8.48e-03	*SPSB2*
rs10420097	19	57633193	G	7.29 (1.82–29.3)	5.18e-03	1.58 (0.14–17.7)	0.712	4.98 (1.49–16.6)	9.13e-03	*ZNF211*
rs3810818	16	4382028	A	2.10 (1.13–3.94)	0.020	1.40 (0.62–3.16)	0.413	1.81 (1.10–2.97)	0.019	*CORO7*
rs2886059	3	126146923	C	2.73 (1.04–7.14)	0.041	1.89 (0.67–5.33)	0.230	2.30 (1.14–4.66)	0.020	*ALDH1L1*
rs9901675	17	7581494	A	4.12 (1.32–12.9)	0.015	1.57 (0.33–7.46)	0.572	2.95 (1.18–7.39)	0.021	*FXR2*
rs4805162	19	36183403	G	2.24 (1.19–4.22)	0.012	1.21 (0.59–2.50)	0.608	1.72 (1.07–2.76)	0.026	*ZNF565*
rs4562	17	7260420	A	2.22 (1.24–3.95)	6.95e-03	1.05 (0.51–2.15)	0.889	1.65 (1.05–2.59)	0.029	*ELP5*
rs17712523	10	71777857	G	2.61 (1.05–6.48)	0.039	1.63 (0.57–4.66)	0.366	2.13 (1.07–4.24)	0.031	*CDH23*
rs2799018	10	31913141	T	1.89 (1.00–3.56)	0.049	1.40 (0.66–2.97)	0.375	1.67 (1.03–2.71)	0.038	*ARHGAP12*
rs12409352	1	160030645	A	2.67 (1.00–7.12)	0.049	1.63 (0.54–4.95)	0.387	2.15 (1.03–4.49)	0.041	*PIGM*
rs4902333	14	64909368	T	2.37 (1.24–4.51)	8.73e-03	0.94 (0.41–2.18)	0.893	1.68 (1.01–2.80)	0.046	*CHURC1*
rs2295490	20	388261	G	2.06 (1.06–4.00)	0.033	1.22 (0.48–3.13)	0.678	1.73 (1.01–2.98)	0.048	*TRIB3*
rs4627790	3	32259860	C	1.98 (1.07–3.68)	0.030	1.13 (0.52–2.45)	0.761	1.59 (0.98–2.59)	0.060	*CMTM8*
rs1048786	16	286916	C	3.55 (1.19–10.6)	0.023	1.15 (0.38–3.48)	0.805	2.03 (0.93–4.43)	0.074	*PDIA2*
rs9901673	17	7580783	A	3.95 (1.79–8.71)	6.53e-04	0.21 (0.054–0.77)	0.019	1.82 (0.92–3.59)	0.084	*SENP3*
rs751655	11	132623600	C	3.08 (1.18–8.02)	0.022	0.99 (0.37–2.62)	0.986	1.76 (0.89–3.49)	0.104	*OPCML*
rs11559290	4	158680524	C	2.08 (1.01–4.28)	0.048	1.03 (0.39–2.74)	0.957	1.62 (0.90–2.90)	0.105	*ETFDH*
rs7858	9	14087770	C	2.89 (1.02–8.19)	0.045	1.12 (0.41–3.09)	0.820	1.78 (0.86–3.67)	0.119	*NFIB*
rs929740	5	73621913	G	2.52 (1.31–4.84)	5.40e-03	0.69 (0.34–1.41)	0.313	1.40 (0.87–2.27)	0.169	*ARHGEF28*
rs9032	9	123104493	C	2.28 (1.07–4.85)	0.033	0.71 (0.25–2.01)	0.515	1.52 (0.82–2.81)	0.179	*STRBP*
rs11552708	17	7559238	A	3.81 (1.50–9.72)	5.05e-03	0.11 (0.020–0.62)	0.012	1.70 (0.75–3.86)	0.207	*SENP3*
rs1061735	7	158733764	G	2.91 (1.10–7.67)	0.031	0.79 (0.32–1.98)	0.618	1.46 (0.75–2.84)	0.265	*ESYT2*
rs11553441	22	42516091	C	3.13 (1.15–8.54)	0.026	0.74 (0.29–1.90)	0.529	1.46 (0.73–2.90)	0.284	*RRP7A*
rs362272	4	3233253	G	2.40 (1.06–5.42)	0.035	0.72 (0.31–1.68)	0.451	1.34 (0.75–2.41)	0.324	*HTT*
rs757978	2	241431686	C	5.18 (1.19–22.6)	0.029	0.74 (0.22–2.42)	0.613	1.59 (0.63–4.01)	0.325	*FARP2*
rs17279558	8	63015187	C	3.61 (1.12–11.7)	0.032	0.37 (0.081–1.74)	0.210	1.56 (0.62–3.97)	0.348	*GGH*
rs3761372	21	39371919	T	3.38 (1.39–8.22)	7.36e-03	0.65 (0.30–1.39)	0.268	1.31 (0.73–2.33)	0.365	*WRB*
rs1061115	10	98417292	G	2.23 (1.13–4.41)	0.021	0.58 (0.28–1.24)	0.161	1.22 (0.74–2.02)	0.438	*PYROXD2*
rs2013169	5	140118020	T	3.32 (1.23–9.01)	0.018	0.28 (0.11–0.73)	9.33e-03	0.92 (0.46–1.84)	0.813	*PURA*

Note: eSNP, single-nucleotide polymorphism associated with cortical expression levels; Chr, gene chromosomal location; OR, odds ratio; CI, confidence interval.

^a,b^All relative to the human reference genome GRCh38 (NCBI Build 38).

^c^Significance threshold for Bonferroni correction at P ≤ 1.52e-03.

## Discussion

To our knowledge, this is the first study investigating the genetic basis of MPH response from an integrative perspective that combines GWAS data, functional annotation, eQTL in relevant tissues to ADHD and pathway enrichment analyses. Our results highlight genes related to nervous system development and function, neurological diseases and psychological disorders. Thus, this comprehensive strategy provides a better understanding of the molecular mechanisms implicated in MPH treatment effects and suggests promising candidates that may contribute to clinical outcome.

In our attempt to improve earlier genetic studies by bridging the gap between genotype and phenotype, we prioritised the nominally significant SNPs based on functional annotation and *cis*-eQTL analysis in human brain, and we identified three candidates previously investigated in ADHD: *ALDH1L1*^34^, *CDH23*^35^ and *CMTM8*^36^. Of these, *CMTM8* showed overlapping association between adult ADHD and bipolar disorder^36^, and *ALDH1L1*, which yielded suggestive results in the present meta-analysis of MPH response, has been related to other neuropsychiatric conditions such as major depressive disorder or schizophrenia^37,38^. In addition, the *ALDH1L1* locus was found among the top hits of a GWAS conducted on children and adolescents with ADHD^34^ and contains non-synonymous rare variants identified through whole-exome sequencing in an ADHD nuclear family^39^. Similarly, *CDH23* harbours one of the top SNPs from a pooling-based GWA of adult ADHD^35^ and reached nominal significance in our meta-analysis. *CDH23* is a member of the cadherin superfamily that mediates calcium-dependent cell-cell adhesion. The activity of cadherins depends on their anchorage to the neuronal cytoskeleton through proteins termed catenins (e.g., CTNNA2), which in turn activate KALRN, a key regulator of dendritic spine development and synaptic plasticity underlying learning and memory^40^. This is of particular interest since catenin-cadherin cell-adhesion complexes are important in cerebellar and hippocampal lamination^41^ and both *CTNNA2* and *KALRN* have shown nominal associations with clinical outcome in our GWAS. In this sense, Park *et al*.^41^ demonstrated that mice lacking the actin-binding domain of Ctnna2 (*cdf*-mutant mice) exhibited abnormal morphology of cerebellum and hippocampus. Moreover, the *cdf*-mutant mice showed an impaired control of the startle response and deficits in startle modulation have also been found in ADHD patients^42,43^. Therefore, cell-adhesion molecules and regulators of synaptic plasticity may play a role in MPH treatment effects, which is in agreement with data from genome-wide linkage and association studies pointing to cadherin13 (*CDH13*) as one of the most consistent candidates implicated in ADHD pathophysiology. Specifically, *CDH13* was detected in three independent GWAS^34,35,44^ and lies within the 16q22-16q24 region identified in a meta-analysis of seven ADHD linkage scans^45^. Furthermore, SNPs in this gene have been linked to defects in verbal working memory and hyperactive/impulsive symptoms in subjects with ADHD^46,47^, addiction vulnerability and drug dependence (e.g., methamphetamine, alcohol, and nicotine)^48,49^.

Pathway enrichment analysis provided further evidence for neuroplastic changes in response to MPH treatment, considering the over-representation of genes involved in morphology of neurons, neuroglia, white matter and brain regions relevant to ADHD (e.g., cerebellum, cerebral cortex, and hippocampus) that we found among eGenes associated with drug response. Our results are in accordance to a wealth of data from neuroimaging studies showing that unmedicated ADHD patients present cortical thickness and reduced white matter volume in several areas of the brain compared to healthy subjects, while medicated children do not differ from control group^50–53^. In addition to structural alterations, ADHD patients exhibit deficits in neural functioning, which may be normalised by MPH. In this sense, Rubia *et al*.^54–56^ demonstrated that MPH restores the aberrant activation and functional connectivity in attention, motivation and interference inhibition networks, as well as during error processing, thus improving neuropsychological performance of subjects with ADHD.

It should also be noted that 15 out of the 32 eGenes included in the pathway enrichment analysis harboured variants which provided preliminary evidence for an association with clinical outcome across the discovery and an independent sample. Our top hit from the meta-analysis, rs17685420, is located in the phosphatidylethanolamine binding protein 4 (PEBP4), a member of an evolutionary conserved family of proteins with pivotal biological functions such as cell proliferation and survival, stimulation of acetylcholine synthesis and inhibition of serine proteases^57^. Given that serine proteases are implicated in many processes during development and tissue homeostasis (e.g., neuronal outgrowth, cell migration, and cell death), disturbances in their activity on the nervous system have been proposed as a possible pathological mechanism for neurological disorders^58^. Indeed, Hohman *et al*.^59^ identified a gene-gene interaction involving *PEBP4* associated with late onset Alzheimer’s disease (AD) across 13 independent datasets, and decreased expression levels have been found in hippocampus of both AD patients and mouse models for another phosphatidylethanolamine binding protein, the PEBP1^60–62^, which has also shown alterations after methamphetamine and morphine administration^63,64^. Additional compelling results were detected for *ARSA*, *SPSB2*, *CORO7* and *PIGM*. The *ARSA* gene encodes the arylsulfatase A, whose deficiency is characterised by decline in school performance, emergence of behavioural problems and neurologic symptoms, such as cerebellar ataxia, among others^65^. *SPSB2* has been associated with borderline personality disorder in a genome-wide methylation analysis^66^ and *CORO7*, which has shown to be important in brain development^67^, was identified as a novel candidate gene for emotionality by comparing the expression profile of two mouse lines with either high or low anxiety-related behaviour^68^. Finally, mutations in the *PIGM* gene, which encodes a protein involved in the synthesis of the glycosylphosphatidylinositol anchor, have been reported in individuals with severe neurological features, including seizures, muscular hypotonia and intellectual disability^69^.

Another interesting finding arising from our research is the significant enrichment for candidates previously related to ADHD or MPH response detected among the set of genes nominally associated with treatment outcome. It is worth mentioning that four of these candidates, namely *CTNNA2* (rs79067553, P = 3.51e-05), *PARD3B* (rs62172701, P = 3.28e-04), *LRP1B* (rs410870, P = 4.00e-04) and *GRM7* (rs17047590, P = 6.36e-04), were significant at P < 1e-03 in the present GWAS analysis. In particular, the metabotropic glutamate receptor 7 (GRM7), which is widely expressed in brain regions relevant to ADHD such as the cerebral cortex, the hippocampus and the cerebellum^51,70^ and has been associated with the disorder^71–73^, was also found among the top hits in a prior GWAS of MPH efficacy^13^, thus supporting the role of the glutaminergic system as a moderator of treatment outcome.

The main strengths of our design include the coverage of a considerably higher number of genetic variants in comparison with the study from Mick *et al*.^13^ (319,722 vs 3,566,199 markers), the use of an integrative approach that combines GWAS data with bioinformatic methods, and the follow up of our top signals in an independent cohort, which did increase the association of a number of markers located in loci with biologically plausible functions (*PEBP4*, *ARSA*, and *SPSB2*). Nevertheless, some limitations should also be considered when interpreting these results. Given the limited sample size, the present study might not be sufficiently powered to detect individual variants of modest effects and we did not identify any loci reaching the genome-wide threshold. This constraint, however, is heavily conditioned on the difficulty to find the required phenotype as shown by the sample size of the studies included in the last meta-analysis of candidate gene-based investigations on MPH response^74^. The small dimension of our paediatric sample could also explain the lack of significance of the PRS derived from the GWAS results in an independent population of ADHD adult patients. Alternatively, this discrepancy may be attributed to differences in the genetic background and the clinical heterogeneity (e.g., comorbidities, frequency of clinical subtypes, and sex ratio) of ADHD among children and adults, as suggested by most of the pharmacogenetic studies conducted in adult samples, which failed to replicate variants previously identified in children and adolescents^75^. Additional methodological aspects or distinct environmental influences between the discovery and the target population may also be responsible for the absence of association.

In conclusion, despite not reaching any genome-wide significant association, our results are consistent with previous findings and highlight genes related to morphological abnormalities in brain regions important for motor control, attention and memory, thus supporting the use of bioinformatic and biological evidence as a complement to GWAS data to disentangle the genetic basis of MPH response.

## Electronic supplementary material

Supplementary Information

Supplementary Table S3

Supplementary Table S4
