# Supplementary material for: Integrative genomic analysis of methylphenidate response in attention-deficit/hyperactivity disorder
Source: Sci Rep. 2018 Jan 30;8:1881. doi: 10.1038/s41598-018-20194-7 (PMC5789875; doi:10.1038/s41598-018-20194-7)
Supplement: Supplementary file 1 — Supplementary Information [file 41598_2018_20194_MOESM1_ESM.doc]

**Supplementary information**

**Integrative genomic analysis of methylphenidate response in attention-deficit/hyperactivity disorder.**

Mireia Pagerols^1,2^, Vanesa Richarte^2-4^, Cristina Sánchez-Mora^1-3^, Paula Rovira^1,2^, María Soler Artigas^1-3^, Iris Garcia-Martínez^1,2^, Eva Calvo-Sánchez^1,2^, Montse Corrales^2,4^, Bruna Santos da Silva^5^, Nina Roth Mota^6,7^, Marcelo Moraes Victor^7^, Luis Augusto Rohde^7,8^, Eugenio Horacio Grevet^7,8^, Claiton Henrique Dotto Bau^5,7^, Bru Cormand^9-12^, Miguel Casas^1-4^, Josep Antoni Ramos-Quiroga^1-4^, *Marta Ribasés^1-3^.

^1^Psychiatric Genetics Unit, Group of Psychiatry, Mental Health and Addiction, Vall d’Hebron Research Institute (VHIR), Universitat Autònoma de Barcelona, Barcelona, Spain.

^2^Department of Psychiatry, Hospital Universitari Vall d’Hebron, Barcelona, Spain.

^3^Biomedical Network Research Centre on Mental Health (CIBERSAM), Instituto de Salud Carlos III, Barcelona, Spain.

^4^Department of Psychiatry and Legal Medicine, Universitat Autònoma de Barcelona, Barcelona, Spain.

^5^Department of Genetics, Institute of Biosciences, Universidade Federal do Rio Grande do Sul, Porto Alegre, Brazil.

^6^Department of Human Genetics and Psychiatry, Donders Institute for Brain, Cognition and Behaviour, Radboud University Medical Centre, Nijmegen, The Netherlands.

^7^ADHD Outpatient Program, Adult Division, Hospital de Clínicas de Porto Alegre, Porto Alegre, Brazil.

^8^Department of Psychiatry, Faculty of Medicine, Universidade Federal do Rio Grande do Sul, Porto Alegre, Brazil.

^9^Departament de Genètica, Microbiologia i Estadística, Facultat de Biologia, Universitat de Barcelona, Barcelona, Spain.

^10^Centro de Investigación Biomédica en Red de Enfermedades Raras (CIBERER), Instituto de Salud Carlos III, Barcelona, Spain.

^11^Institut de Biomedicina de la Universitat de Barcelona (IBUB), Barcelona, Spain.

^12^Institut de Recerca Sant Joan de Déu (IR-SJD), Esplugues de Llobregat, Spain

*Correspondence: Marta Ribasés, Psychiatric Genetics Unit, Vall d’Hebron Research Institute, Passeig Vall d’Hebron 119-129, 08035 Barcelona, Spain; e-mail: marta.ribases@vhir.org

**Supplementary Table S1** Candidate genes for ADHD and/or methylphenidate response based on genetic and pharmacogenetic studies

| **Gene symbol^a^** | **Official name^b^** | **Location^c^** | **Studied phenotype** | **References** |
| --- | --- | --- | --- | --- |
| *AANAT* | aralkylamine N-acetyltransferase | 17q25.1 | ADHD | [^1^](#_ENREF_1) |
| *ABCB1* | ATP binding cassette subfamily B member 1 | 7q21.12 | MPH response | [^2^](#_ENREF_2) |
| *ADGRA3* | adhesion G protein-coupled receptor A3 | 4p15.2 | ADHD | [^3^](#_ENREF_3) |
| *ADGRL3* | adhesion G protein-coupled receptor L3 | 4q13.1 | ADHD and MPH response | [^4-10^](#_ENREF_4) |
| *ADRA1A* | adrenoceptor alpha 1A | 8p21.2 | ADHD | [^11-13^](#_ENREF_11) |
| *ADRA1B* | adrenoceptor alpha 1B | 5q33.3 | ADHD |  |
| *ADRA1D* | adrenoceptor alpha 1D | 20p13 | ADHD | [^13^](#_ENREF_13) |
| *ADRA2A* | adrenoceptor alpha 2A | 10q25.2 | ADHD and MPH response | [^14-20^](#_ENREF_14); For a review of ADHD genetic studies, see [^21^](#_ENREF_21) |
| *ADRA2B* | adrenoceptor alpha 2B | 2q11.2 | ADHD | [^13^](#_ENREF_13) |
| *ADRA2C* | adrenoceptor alpha 2C | 4p16.3 | ADHD |  |
| *ADRB1* | adrenoceptor beta 1 | 10q25.3 | ADHD |  |
| *ADRB2* | adrenoceptor beta 2 | 5q32 | ADHD |  |
| *ADRB3* | adrenoceptor beta 3 | 8p11.23 | ADHD |  |
| *AGBL1* | ATP/GTP binding protein like 1 | 15q25.3 | ADHD | [^26^](#_ENREF_26) |
| *AK8* | adenylate kinase 8 | 9q34.13 | ADHD | [^27^](#_ENREF_27) |
| *ALDH1L1* | aldehyde dehydrogenase 1 family member L1 | 3q21.3 | ADHD |  |
| *ANK3* | ankyrin 3 | 10q21.2 | ADHD |  |
| *ANKK1* | ankyrin repeat and kinase domain containing 1 | 11q23.2 | ADHD and MPH response |  |
| *ANO5* | anoctamin 5 | 11p14.3 | ADHD | [^34^](#_ENREF_34) |
| *ARRB1* | arrestin beta 1 | 11q13.4 | ADHD |  |
| *ARRB2* | arrestin beta 2 | 17p13.2 | ADHD | [^12^](#_ENREF_12) |
| *ARSB* | arylsulfatase B | 5q14.1 | ADHD | [^35^](#_ENREF_35) |
| *ARVCF* | armadillo repeat gene deleted in velocardiofacial syndrome | 22q11.21 | ADHD | [^36^](#_ENREF_36) |
| *AS3MT* | arsenite methyltransferase | 10q24.32 | ADHD | [^37^](#_ENREF_37) |
| *ASMT* | acetylserotonin O-methyltransferase | Xp22.33 and Yp11.2 | ADHD | [^1^](#_ENREF_1) |
| *ASTN2* | astrotactin 2 | 9q33.1 | ADHD |  |
| *ATP11A* | ATPase phospholipid transporting 11A | 13q34 | ADHD | [^29^](#_ENREF_29) |
| *ATP2B3* | ATPase plasma membrane Ca2+ transporting 3 | Xq28 | ADHD | [^39^](#_ENREF_39) |
| *ATP2C2* | ATPase secretory pathway Ca2+ transporting 2 | 16q24.1 | ADHD |  |
| *ATP7B* | ATPase copper transporting beta | 13q14.3 | ADHD | [^28^](#_ENREF_28) |
| *ATXN1* | ataxin 1 | 6p22.3 | ADHD | [^26^](#_ENREF_26) |
| *ATXN2* | ataxin 2 | 12q24.12 | ADHD | [^40^](#_ENREF_40) |
| *AUTS2* | autism susceptibility candidate 2 | 7q11.22 | ADHD |  |
| *BAALC-AS2* | BAALC antisense RNA 2 | 8q22.3 | ADHD | [^43^](#_ENREF_43) |
| *BAIAP2* | BAI1 associated protein 2 | 17q25.3 | ADHD | [^39^](#_ENREF_39) |
| *BCHE* | butyrylcholinesterase | 3q26.1 | ADHD | [^44^](#_ENREF_44) |
| *BCL11A* | B-cell CLL/lymphoma 11A | 2p16.1 | ADHD | [^3^](#_ENREF_3) |
| *BDNF* | brain derived neurotrophic factor | 11p14.1 | ADHD and MPH response | [^45^](#_ENREF_45); For a review of ADHD genetic studies, see [^21^](#_ENREF_21) |
| *BMPR1B* | bone morphogenetic protein receptor type 1B | 4q22.3 | ADHD | [^34^](#_ENREF_34) |
| *BRE* | brain and reproductive organ-expressed (TNFRSF1A modulator) | 2p23.2 | ADHD | [^30^](#_ENREF_30) |
| *CACNA1A* | calcium voltage-gated channel subunit alpha1 A | 19p13.13 | ADHD | [^46^](#_ENREF_46) |
| *CACNA1C* | calcium voltage-gated channel subunit alpha1 C | 12p13.33 | ADHD |  |
| *CACNA1D* | calcium voltage-gated channel subunit alpha1 D | 3p21.1 | ADHD | [^46^](#_ENREF_46) |
| *CACNB2* | calcium voltage-gated channel auxiliary subunit beta 2 | 10p12.33-p12.31 | ADHD | [^37^](#_ENREF_37) |
| *CADM2* | cell adhesion molecule 2 | 3p12.1 | ADHD | [^47^](#_ENREF_47) |
| *CALY* | calcyon neuron specific vesicular protein | 10q26.3 | ADHD |  |
| *CAMK1D* | calcium/calmodulin dependent protein kinase ID | 10p13 | ADHD | [^26^](#_ENREF_26) |
| *CARTPT* | CART prepropeptide | 5q13.2 | ADHD | [^49^](#_ENREF_49) |
| *CCDC192* | coiled-coil domain containing 192 | 5q23.2-q23.3 | ADHD | [^43^](#_ENREF_43) |
| *CCSER1* | coiled-coil serine rich protein 1 | 4q22.1 | ADHD | [^50^](#_ENREF_50) |
| *CDH13* | cadherin 13 | 16q23.3 | ADHD |  |
| *CDH23* | cadherin related 23 | 10q22.1 | ADHD | [^27^](#_ENREF_27) |
| *CDH26* | cadherin 26 | 20q13.33 | ADHD | [^26^](#_ENREF_26) |
| *CDK20* | cyclin dependent kinase 20 | 9q22.1 | ADHD | [^3^](#_ENREF_3) |
| *CEP112* | centrosomal protein 112 | 17q24.1 | ADHD | [^34^](#_ENREF_34) |
| *CES1* | carboxylesterase 1 | 16q12.2 | MPH response | [^54^](#_ENREF_54) |
| *CFAP221* | cilia and flagella associated protein 221 | 2q14.2 | ADHD | [^27^](#_ENREF_27) |
| *CFAP43* | cilia and flagella associated protein 43 | 10q25.1 | ADHD | [^27^](#_ENREF_27) |
| *CHMP7* | charged multivesicular body protein 7 | 8p21.3 | ADHD | [^26^](#_ENREF_26) |
| *CHRNA3* | cholinergic receptor nicotinic alpha 3 subunit | 15q25.1 | ADHD | [^55^](#_ENREF_55) |
| *CHRNA4* | cholinergic receptor nicotinic alpha 4 subunit | 20q13.33 | ADHD and MPH response |  |
| *CHRNA7* | cholinergic receptor nicotinic alpha 7 subunit | 15q13.3 | ADHD |  |
| *CLASP2* | cytoplasmic linker associated protein 2 | 3p22.3 | ADHD | [^3^](#_ENREF_3) |
| *CLOCK* | clock circadian regulator | 4q12 | ADHD |  |
| *CLYBL* | citrate lyase beta like | 13q32.3 | ADHD | [^53^](#_ENREF_53) |
| *CMTM8* | CKLF like MARVEL transmembrane domain containing 8 | 3p22.3 | ADHD | [^64^](#_ENREF_64) |
| *CNMD* | chondromodulin | 13q14.3 | ADHD | [^34^](#_ENREF_34) |
| *CNR1* | cannabinoid receptor 1 | 6q15 | ADHD | [^65^](#_ENREF_65) |
| *CNTF* | ciliary neurotrophic factor | 11q12.1 | ADHD | [^66^](#_ENREF_66) |
| *CNTFR* | ciliary neurotrophic factor receptor | 9p13.3 | ADHD | [^66^](#_ENREF_66) |
| *CNTN4* | contactin 4 | 3p26.3-p26.2 | ADHD | [^67^](#_ENREF_67) |
| *CNTN5* | contactin 5 | 11q22.1 | ADHD | [^38^](#_ENREF_38) |
| *CNTNAP2* | contactin associated protein-like 2 | 7q35-q36.1 | ADHD | [^42^](#_ENREF_42) |
| *CNTNAP4* | contactin associated protein like 4 | 16q23.1 | ADHD |  |
| *CNTNAP5* | contactin associated protein like 5 | 2q14.3 | ADHD | [^53^](#_ENREF_53) |
| *COMT* | catechol-O-methyltransferase | 22q11.21 | ADHD and MPH response | ; For a review of ADHD genetic studies, see [^21^](#_ENREF_21) |
| *COX7B2* | cytochrome c oxidase subunit 7B2 | 4p12 | ADHD | [^3^](#_ENREF_3) |
| *CPLX1* | complexin 1 | 4p16.3 | ADHD | [^74^](#_ENREF_74) |
| *CPLX2* | complexin 2 | 5q35.2 | ADHD |  |
| *CPLX4* | complexin 4 | 18q21.32 | ADHD | [^74^](#_ENREF_74) |
| *CREB5* | cAMP responsive element binding protein 5 | 7p15.1-p14.3 | ADHD | [^27^](#_ENREF_27) |
| *CRYGC* | crystallin gamma C | 2q33.3 | ADHD | [^27^](#_ENREF_27) |
| *CSMD2* | CUB and Sushi multiple domains 2 | 1p35.1 | ADHD | [^27^](#_ENREF_27) |
| *CSNK1E* | casein kinase 1 epsilo | 22q13.1 | ADHD |  |
| *CSTF2T* | cleavage stimulation factor subunit 2 tau variant | 10q21.1 | ADHD |  |
| *CTNNA2* | catenin alpha 2 | 2p12 | ADHD |  |
| *DACT1* | dishevelled binding antagonist of beta catenin 1 | 14q23.1 | ADHD | [^39^](#_ENREF_39) |
| *DBH* | dopamine beta-hydroxylase | 9q34.2 | ADHD and MPH response | ; For a review of ADHD genetic studies, see [^21^](#_ENREF_21) |
| *DCDC2* | doublecortin domain containing 2 | 6p22.3 | ADHD | [^76^](#_ENREF_76) |
| *DCLK1* | doublecortin like kinase 1 | 13q13.3 | ADHD | [^77^](#_ENREF_77) |
| *DCLK2* | doublecortin like kinase 2 | 4q31.23-q31.3 | ADHD | [^38^](#_ENREF_38) |
| *DDC* | dopa decarboxylase | 7p12.2-p12.1 | ADHD |  |
| *DENND3* | DENN domain containing 3 | 8q24.3 | ADHD | [^3^](#_ENREF_3) |
| *DGKH* | diacylglycerol kinase eta | 13q14.11 | ADHD |  |
| *DHCR7* | 7-dehydrocholesterol reductase | 11q13.4 | ADHD | [^26^](#_ENREF_26) |
| *DIRAS2* | DIRAS family GTPase 2 | 9q22.2 | ADHD | [^82^](#_ENREF_82) |
| *DISC1* | disrupted in schizophrenia 1 | 1q42.2 | ADHD | [^83^](#_ENREF_83) |
| *DLEU2* | deleted in lymphocytic leukemia 2 (non-protein coding) | 13q14.2 | ADHD | [^40^](#_ENREF_40) |
| *DMRT2* | doublesex and mab-3 related transcription factor 2 | 9p24.3 | ADHD | [^53^](#_ENREF_53) |
| *DNAJA1P4* | DnaJ heat shock protein family (Hsp40) member A1 pseudogene 4 | 6q22.1 | ADHD | [^34^](#_ENREF_34) |
| *DNAJC27* | DnaJ heat shock protein family (Hsp40) member C27 | 2p23.3 | ADHD | [^47^](#_ENREF_47) |
| *DNM1* | dynamin 1 | 9q34.11 | ADHD | [^27^](#_ENREF_27) |
| *DNMT3B* | DNA methyltransferase 3 beta | 20q11.21 | ADHD | [^43^](#_ENREF_43) |
| *DOCK10* | dedicator of cytokinesis 10 | 2q36.2 | ADHD | [^40^](#_ENREF_40) |
| *DPH6* | diphthamine biosynthesis 6 | 15q14 | ADHD | [^34^](#_ENREF_34) |
| *DPP10* | dipeptidyl peptidase like 10 | 2q14.1 | ADHD |  |
| *DPP6* | dipeptidyl peptidase like 6 | 7q36.2 | ADHD |  |
| *DRD1* | dopamine receptor D1 | 5q35.2 | ADHD and MPH response |  |
| *DRD2* | dopamine receptor D2 | 11q23.2 | ADHD and MPH response |  |
| *DRD3* | dopamine receptor D3 | 3q13.31 | ADHD and MPH response |  |
| *DRD4* | dopamine receptor D4 | 11p15.5 | ADHD and MPH response | For a review of ADHD genetic and pharmacogenetic studies, see [^21^](#_ENREF_21) and , respectively |
| *DRD5* | dopamine receptor D5 | 4p16.1 | ADHD and MPH response | ; For a review of ADHD genetic studies, see [^21^](#_ENREF_21) |
| *DSCC1* | DNA replication and sister chromatid cohesion 1 | 8q24.12 | ADHD | [^35^](#_ENREF_35) |
| *DUSP1* | dual specificity phosphatase 1 | 5q35.1 | ADHD | [^34^](#_ENREF_34) |
| *DYX1C1* | dyslexia susceptibility 1 candidate 1 | 15q21.3 | ADHD | [^102^](#_ENREF_102) |
| *EGFR* | epidermal growth factor receptor | 7p11.2 | ADHD | [^64^](#_ENREF_64) |
| *ELOC* | elongin C | 8q21.11 | ADHD | [^26^](#_ENREF_26) |
| *ELOVL6* | ELOVL fatty acid elongase 6 | 4q25 | ADHD | [^34^](#_ENREF_34) |
| *EMP2* | epithelial membrane protein 2 | 16p13.13 | ADHD |  |
| *EREG* | epiregulin | 4q13.3 | ADHD | [^53^](#_ENREF_53) |
| *ERICH3* | glutamate rich 3 | 1p31.1 | ADHD | [^29^](#_ENREF_29) |
| *ETV5* | ETS variant 5 | 3q27.2 | ADHD | [^47^](#_ENREF_47) |
| *FADS1* | fatty acid desaturase 1 | 11q12.2 | ADHD |  |
| *FADS2* | fatty acid desaturase 2 | 11q12.2 | ADHD |  |
| *FAIM2* | Fas apoptotic inhibitory molecule 2 | 12q13.12 | ADHD | [^47^](#_ENREF_47) |
| *FAM189A1* | family with sequence similarity 189 member A1 | 15q13.1 | ADHD |  |
| *FANCL* | Fanconi anemia complementation group L | 2p16.1 | ADHD | [^47^](#_ENREF_47) |
| *FBXO33* | F-box protein 33 | 14q21.1 | ADHD | [^104^](#_ENREF_104) |
| *FGF10* | fibroblast growth factor 10 | 5p12 | ADHD | [^105^](#_ENREF_105) |
| *FGF12* | fibroblast growth factor 12 | 3q28-q29 | ADHD | [^30^](#_ENREF_30) |
| *FHIT* | fragile histidine triad | 3p14.2 | ADHD | [^53^](#_ENREF_53) |
| *FLNC* | filamin C | 7q32.1 | ADHD | [^29^](#_ENREF_29) |
| *FLRT2* | fibronectin leucine rich transmembrane protein 2 | 14q31.3 | ADHD | [^35^](#_ENREF_35) |
| *FOXP1* | forkhead box P1 | 3p13 | ADHD | [^53^](#_ENREF_53) |
| *FOXP2* | forkhead box P2 | 7q31.1 | ADHD | [^106^](#_ENREF_106) |
| *FRMD1* | FERM domain containing 1 | 6q27 | ADHD | [^43^](#_ENREF_43) |
| *FTO* | FTO, alpha-ketoglutarate dependent dioxygenase | 16q12.2 | ADHD |  |
| *FURIN* | urin, paired basic amino acid cleaving enzyme | 15q26.1 | ADHD | [^26^](#_ENREF_26) |
| *GABRG1* | gamma-aminobutyric acid type A receptor gamma1 subunit | 4p12 | ADHD | [^38^](#_ENREF_38) |
| *GDNF* | glial cell derived neurotrophic factor | 5p13.2 | ADHD |  |
| *GEMIN2* | gem nuclear organelle associated protein 2 | 14q21.1 | ADHD | [^30^](#_ENREF_30) |
| *GFI1B* | growth factor independent 1B transcriptional repressor | 9q34.13 | ADHD | [^3^](#_ENREF_3) |
| *GFOD1* | glucose-fructose oxidoreductase domain containing 1 | 6p24.1-p23 | ADHD | [^53^](#_ENREF_53) |
| *GIT1* | GIT ArfGAP 1 | 17q11.2 | ADHD |  |
| *GNAL* | G protein subunit alpha L | 18p11.21 | ADHD | [^111^](#_ENREF_111) |
| *GNAO1* | G protein subunit alpha o1 | 16q13 | ADHD | [^112^](#_ENREF_112) |
| *GNAT2* | G protein subunit alpha transducin 2 | 1p13.3 | ADHD | [^112^](#_ENREF_112) |
| *GNAZ* | G protein subunit alpha z | 22q11.22-q11.23 | ADHD | [^112^](#_ENREF_112) |
| *GNPDA2* | glucosamine-6-phosphate deaminase 2 | 4p12 | ADHD | [^47^](#_ENREF_47) |
| *GPC5* | glypican 5 | 13q31.3 | ADHD | [^3^](#_ENREF_3) |
| *GPC6* | glypican 6 | 13q31.3-q32.1 | ADHD | [^27^](#_ENREF_27) |
| *GPR139* | G protein-coupled receptor 139 | 16p12.3 | ADHD |  |
| *GPR50* | G protein-coupled receptor 50 | Xq28 | ADHD | [^1^](#_ENREF_1) |
| *GPRC5B* | G protein-coupled receptor class C group 5 member B | 16p12.3 | ADHD | [^47^](#_ENREF_47) |
| *GPX6* | glutathione peroxidase 6 | 6p22.1 | ADHD | [^3^](#_ENREF_3) |
| *GRID2* | glutamate ionotropic receptor delta type subunit 2 | 4q22.1-q22.2 | ADHD | [^40^](#_ENREF_40) |
| *GRIK1* | glutamate ionotropic receptor kainate type subunit 1 | 21q21.3 | ADHD | [^53^](#_ENREF_53) |
| *GRIK4* | glutamate ionotropic receptor kainate type subunit 4 | 11q23.3 | ADHD |  |
| *GRIN2A* | glutamate ionotropic receptor NMDA type subunit 2A | 16p13.2 | ADHD |  |
| *GRIN2B* | glutamate ionotropic receptor NMDA type subunit 2B | 12p13.1 | ADHD |  |
| *GRK2* | G protein-coupled receptor kinase 2 | 11q13.2 | ADHD | [^12^](#_ENREF_12) |
| *GRK3* | G protein-coupled receptor kinase 3 | 22q12.1 | ADHD | [^12^](#_ENREF_12) |
| *GRM1* | glutamate metabotropic receptor 1 | 6q24.3 | ADHD | [^67^](#_ENREF_67) |
| *GRM5* | glutamate metabotropic receptor 5 | 11q14.2-q14.3 | ADHD |  |
| *GRM7* | glutamate metabotropic receptor 7 | 3p26.1 | ADHD and MPH response |  |
| *GRM8* | glutamate metabotropic receptor 8 | 7q31.33 | ADHD | [^67^](#_ENREF_67) |
| *GSK3B* | glycogen synthase kinase 3 beta | 3q13.33 | ADHD | [^119^](#_ENREF_119) |
| *GUCY1A2* | guanylate cyclase 1 soluble subunit alpha 2 | 11q22.3 | ADHD |  |
| *H2AFY* | H2A histone family member Y | 5q31.1 | ADHD | [^3^](#_ENREF_3) |
| *HAS3* | hyaluronan synthase 3 | 16q22.1 | ADHD | [^53^](#_ENREF_53) |
| *HCN1* | hyperpolarization activated cyclic nucleotide gated potassium channel 1 | 5p12 | ADHD | [^105^](#_ENREF_105) |
| *HECTD2-AS1* | HECTD2 antisense RNA 1 | 10q23.32 | ADHD | [^55^](#_ENREF_55) |
| *HES1* | hes family bHLH transcription factor 1 | 3q29 | ADHD | [^12^](#_ENREF_12) |
| *HES6* | hes family bHLH transcription factor 6 | 2q37.3 | ADHD | [^12^](#_ENREF_12) |
| *HK1* | hexokinase 1 | 10q22.1 | ADHD | [^29^](#_ENREF_29) |
| *HKDC1* | hexokinase domain containing 1 | 10q22.1 | ADHD | [^29^](#_ENREF_29) |
| *HLA-DRB1* | major histocompatibility complex, class II, DR beta 1 | 6p21.32 | ADHD |  |
| *HOXB1* | homeobox B | 17q21.32 | ADHD | [^40^](#_ENREF_40) |
| *HTR1A* | 5-hydroxytryptamine receptor 1A | 5q12.3 | ADHD |  |
| *HTR1B* | 5-hydroxytryptamine receptor 1B | 6q14.1 | ADHD and MPH response | ; For a review of ADHD genetic studies, see [^21^](#_ENREF_21) |
| *HTR1D* | 5-hydroxytryptamine receptor 1D | 1p36.12 | ADHD |  |
| *HTR1E* | 5-hydroxytryptamine receptor 1E | 6q14.3 | ADHD |  |
| *HTR1F* | 5-hydroxytryptamine receptor 1F | 3p11.2-p11.1 | ADHD | [^78^](#_ENREF_78) |
| *HTR2A* | 5-hydroxytryptamine receptor 2A | 13q14.2 | ADHD and MPH response | [^126^](#_ENREF_126); For a review of ADHD genetic studies, see [^21^](#_ENREF_21) |
| *HTR2B* | 5-hydroxytryptamine receptor 2B | 2q37.1 | ADHD | [^78^](#_ENREF_78) |
| *HTR2C* | 5-hydroxytryptamine receptor 2C | Xq23 | ADHD |  |
| *HTR3A* | 5-hydroxytryptamine receptor 3A | 11q23.2 | ADHD | [^78^](#_ENREF_78) |
| *HTR3B* | 5-hydroxytryptamine receptor 3B | 11q23.2 | ADHD |  |
| *HTR4* | 5-hydroxytryptamine receptor 4 | 5q32 | ADHD |  |
| *HTR5A* | 5-hydroxytryptamine receptor 5A | 7q36.2 | ADHD |  |
| *HTR6* | 5-hydroxytryptamine receptor 6 | 1p36.13 | ADHD |  |
| *HTR7* | 5-hydroxytryptamine receptor 7 | 10q23.31 | ADHD | [^78^](#_ENREF_78) |
| *ID2* | inhibitor of DNA binding 2, HLH protein | 2p25.1 | ADHD | [^78^](#_ENREF_78) |
| *IL16* | interleukin 16 | 15q25.1 | ADHD | [^53^](#_ENREF_53) |
| *IL1RN* | interleukin 1 receptor antagonist | 2q14.1 | ADHD |  |
| *IL20RA* | interleukin 20 receptor subunit alpha | 6q23.3 | ADHD | [^40^](#_ENREF_40) |
| *IMMP2L* | inner mitochondrial membrane peptidase subunit 2 | 7q31.1 | ADHD | [^42^](#_ENREF_42) |
| *ISL1* | ISL LIM homeobox 1 | 5q11.1 | ADHD | [^105^](#_ENREF_105) |
| *ITGA1* | integrin subunit alpha 1 | 5q11.2 | ADHD |  |
| *ITGA11* | integrin subunit alpha 11 | 15q23 | ADHD | [^27^](#_ENREF_27) |
| *ITGAE* | integrin subunit alpha E | 17p13.2 | ADHD | [^27^](#_ENREF_27) |
| *ITIH3* | inter-alpha-trypsin inhibitor heavy chain 3 | 3p21.1 | ADHD | [^37^](#_ENREF_37) |
| *KALRN* | kalirin, RhoGEF kinase | 3q21.1-q21.2 | ADHD | [^27^](#_ENREF_27) |
| *KANK2* | KN motif and ankyrin repeat domains 2 | 19p13.2 | ADHD | [^3^](#_ENREF_3) |
| *KANSL1* | KAT8 regulatory NSL complex subunit 1 | 17q21.31 | ADHD | [^3^](#_ENREF_3) |
| *KCNC1* | potassium voltage-gated channel subfamily C member 1 | 11p15.1 | ADHD | [^27^](#_ENREF_27) |
| *KCNIP1* | potassium voltage-gated channel interacting protein 1 | 5q35.1 | ADHD |  |
| *KCNIP4* | potassium voltage-gated channel interacting protein 4 | 4p15.31-p15.2 | ADHD | [^134^](#_ENREF_134) |
| *KCTD15* | potassium channel tetramerization domain containing 15 | 19q13.11 | ADHD | [^47^](#_ENREF_47) |
| *KIAA0319* | KIAA0319 | 6p22.3 | ADHD | [^76^](#_ENREF_76) |
| *LARGE1* | LARGE xylosyl- and glucuronyltransferase 1 | 22q12.3 | ADHD | [^53^](#_ENREF_53) |
| *LARP7* | La ribonucleoprotein domain family member 7 | 4q25 | ADHD | [^67^](#_ENREF_67) |
| *LHFPL3* | lipoma HMGIC fusion partner-like 3 | 7q22.2-q22.3 | ADHD | [^30^](#_ENREF_30) |
| *LIN7C* | lin-7 homolog C, crumbs cell polarity complex component | 11p14.1 | ADHD | [^135^](#_ENREF_135) |
| *LINC00494* | long intergenic non-protein coding RNA 494 | 20q13.13 | ADHD | [^43^](#_ENREF_43) |
| *LINGO2* | leucine rich repeat and Ig domain containing 2 | 9p21.2-p21.1 | ADHD | [^47^](#_ENREF_47) |
| *LMAN2L* | lectin, mannose binding 2 like | 2q11.2 | ADHD | [^30^](#_ENREF_30) |
| *LMO4* | LIM domain only 4 | 1p22.3 | ADHD | [^39^](#_ENREF_39) |
| *LOC100287010* | uncharacterized LOC100287010 | 2q12.1 | ADHD | [^43^](#_ENREF_43) |
| *LOC100506534* | uncharacterized LOC100506534 | 7 | ADHD | [^53^](#_ENREF_53) |
| *LOC105370982* | uncharacterized LOC105370982 | 15q26.1 | ADHD | [^27^](#_ENREF_27) |
| *LOC151121* | uncharacterized LOC151121 | 2q21.1 | ADHD | [^3^](#_ENREF_3) |
| *LOC392232* | transient receptor potential cation channel subfamily A member 1 pseudogene | 8q21.11 | ADHD | [^43^](#_ENREF_43) |
| *LOC643542* | uncharacterized LOC643542 | 18q22.1 | ADHD | [^53^](#_ENREF_53) |
| *LOXL2* | lysyl oxidase like 2 | 8p21.3 | ADHD | [^26^](#_ENREF_26) |
| *LPL* | lipoprotein lipase | 8p21.3 | ADHD | [^53^](#_ENREF_53) |
| *LRP1B* | LDL receptor related protein 1B | 2q22.1-q22.2 | ADHD | [^47^](#_ENREF_47) |
| *LRRC7* | leucine rich repeat containing 7 | 1p31.1 | ADHD | [^53^](#_ENREF_53) |
| *MACROD2* | MACRO domain containing 2 | 20p12.1 | ADHD | [^38^](#_ENREF_38) |
| *MAD1L1* | MAD1 mitotic arrest deficient like 1 | 7p22.3 | ADHD | [^30^](#_ENREF_30) |
| *MAGI2* | membrane associated guanylate kinase, WW and PDZ domain containing 2 | 7q21.11 | ADHD | [^40^](#_ENREF_40) |
| *MAN2A2* | mannosidase alpha class 2A member 2 | 15q26.1 | ADHD | [^27^](#_ENREF_27) |
| *MAOA* | monoamine oxidase A | Xp11.3 | ADHD and MPH response | [^136^](#_ENREF_136); For a review of ADHD genetic studies, see [^21^](#_ENREF_21) |
| *MAOB* | monoamine oxidase B | Xp11.3 | ADHD |  |
| *MAP1B* | microtubule associated protein 1B | 5q13.2 | ADHD | [^27^](#_ENREF_27) |
| *MAP2K3* | mitogen-activated protein kinase kinase 3 | 17p11.2 | ADHD | [^3^](#_ENREF_3) |
| *MAP2K5* | mitogen-activated protein kinase kinase 5 | 15q23 | ADHD | [^47^](#_ENREF_47) |
| *MAPRE1* | microtubule associated protein RP/EB family member 1 | 20q11.21 | ADHD | [^43^](#_ENREF_43) |
| *MBOAT1* | membrane bound O-acyltransferase domain containing 1 | 6p22.3 | ADHD | [^53^](#_ENREF_53) |
| *MC4R* | melanocortin 4 receptor | 18q21.32 | ADHD | [^47^](#_ENREF_47) |
| *MCTP1* | multiple C2 and transmembrane domain containing 1 | 5q15 | ADHD | [^34^](#_ENREF_34) |
| *MED27* | mediator complex subunit 27 | 9q34.13 | ADHD | [^35^](#_ENREF_35) |
| *MEIS2* | Meis homeobox 2 | 15q14 | ADHD | [^53^](#_ENREF_53) |
| *METTL3* | methyltransferase like 3 | 14q11.2 | ADHD | [^28^](#_ENREF_28) |
| *MIR96* | microRNA 96 | 7q32.2 | ADHD | [^141^](#_ENREF_141) |
| *MIR99AHG* | mir-99a-let-7c cluster host gene | 21q21.1 | ADHD | [^34^](#_ENREF_34) |
| *MMP24* | matrix metallopeptidase 24 | 20q11.22 | ADHD | [^27^](#_ENREF_27) |
| *MMP7* | matrix metallopeptidase 7 | 11q22.2 | ADHD | [^3^](#_ENREF_3) |
| *MOBP* | myelin-associated oligodendrocyte basic protein | 3p22.1 | ADHD | [^27^](#_ENREF_27) |
| *MOG* | myelin oligodendrocyte glycoprotein | 6p22.1 | ADHD | [^142^](#_ENREF_142) |
| *MTA3* | metastasis associated 1 family member 3 | 2p21 | ADHD | [^53^](#_ENREF_53) |
| *MTCH2* | mitochondrial carrier 2 | 11p11.2 | ADHD | [^47^](#_ENREF_47) |
| *MTHFR* | methylenetetrahydrofolate reductase | 1p36.22 | ADHD | [^143^](#_ENREF_143) |
| *MTIF3* | mitochondrial translational initiation factor 3 | 13q12.2 | ADHD | [^47^](#_ENREF_47) |
| *MTNR1A* | melatonin receptor 1A | 4q35.2 | ADHD | [^1^](#_ENREF_1) |
| *MTNR1B* | melatonin receptor 1B | 11q14.3 | ADHD | [^1^](#_ENREF_1) |
| *MYBPC1* | myosin binding protein C, slow type | 12q23.2 | ADHD | [^3^](#_ENREF_3) |
| *MYO5B* | myosin VB | 18q21.1 | ADHD | [^31^](#_ENREF_31) |
| *MYT1L* | myelin transcription factor 1 like | 2p25.3 | ADHD | [^27^](#_ENREF_27) |
| *NADSYN1* | NAD synthetase 1 | 11q13.4 | ADHD | [^26^](#_ENREF_26) |
| *NAPRT* | nicotinate phosphoribosyltransferase | 8q24.3 | ADHD | [^53^](#_ENREF_53) |
| *NCAM1* | neural cell adhesion molecule 1 | 11q23.2 | ADHD and MPH response |  |
| *NCAN* | neurocan | 19p13.11 | ADHD | [^30^](#_ENREF_30) |
| *NCKAP5* | NCK associated protein 5 | 2q21.2 | ADHD | [^53^](#_ENREF_53) |
| *NCL* | nucleolin | 2q37.1 | ADHD | [^35^](#_ENREF_35) |
| *NDN* | necdin, MAGE family member | 15q11.2 | ADHD |  |
| *NEGR1* | neuronal growth regulator 1 | 1p31.1 | ADHD |  |
| *NEUROD6* | neuronal differentiation 6 | 7p14.3 | ADHD | [^39^](#_ENREF_39) |
| *NFIL3* | nuclear factor, interleukin 3 regulated | 9q22.31 | ADHD |  |
| *NGF* | nerve growth factor | 1p13.2 | ADHD |  |
| *NGFR* | nerve growth factor receptor | 17q21.33 | ADHD | [^66^](#_ENREF_66) |
| *NLN* | neurolysin | 5q12.3 | ADHD | [^67^](#_ENREF_67) |
| *NOS1* | nitric oxide synthase 1 | 12q24.22 | ADHD | [^144-146^](#_ENREF_144) |
| *NOS3* | nitric oxide synthase 3 | 7q36.1 | ADHD | [^145^](#_ENREF_145) |
| *NPAS3* | neuronal PAS domain protein 3 | 14q13.1 | ADHD | [^64^](#_ENREF_64) |
| *NPPC* | natriuretic peptide C | 2q37.1 | ADHD | [^3^](#_ENREF_3) |
| *NPSR1* | neuropeptide S receptor 1 | 7p14.3 | ADHD | [^147^](#_ENREF_147) |
| *NPY* | neuropeptide Y | 7p15.3 | ADHD |  |
| *NR3C2* | nuclear receptor subfamily 3 group C member 2 | 4q31.23 | ADHD | [^150^](#_ENREF_150) |
| *NR4A2* | nuclear receptor subfamily 4 group A member 2 | 2q24.1 | ADHD |  |
| *NRSN1* | neurensin 1 | 6p22.3 | ADHD | [^76^](#_ENREF_76) |
| *NRXN1* | neurexin 1 | 2p16.3 | ADHD | [^29^](#_ENREF_29) |
| *NRXN3* | neurexin 3 | 14q24.3-q31.1 | ADHD | [^47^](#_ENREF_47) |
| *NSF* | N-ethylmaleimide sensitive factor, vesicle fusing ATPase | 17q21.31 | ADHD | [^74^](#_ENREF_74) |
| *NT5C2* | 5'-nucleotidase, cytosolic II | 10q24.32-q24.33 | ADHD | [^37^](#_ENREF_37) |
| *NT5DC3* | 5'-nucleotidase domain containing 3 | 12q23.3 | ADHD | [^27^](#_ENREF_27) |
| *NTF3* | neurotrophin 3 | 12p13.31 | ADHD and MPH response |  |
| *NTF4* | neurotrophin 4 | 19q13.33 | ADHD | [^66^](#_ENREF_66) |
| *NTM* | neurotrimin | 11q25 | ADHD | [^35^](#_ENREF_35) |
| *NTRK1* | neurotrophic receptor tyrosine kinase 1 | 1q23.1 | ADHD | [^66^](#_ENREF_66) |
| *NTRK2* | neurotrophic receptor tyrosine kinase 2 | 9q21.33 | ADHD | [^66^](#_ENREF_66) |
| *NTRK3* | neurotrophic receptor tyrosine kinase 3 | 15q25.3 | ADHD | [^66^](#_ENREF_66) |
| *NUCB1* | nucleobindin 1 | 19q13.33 | ADHD | [^26^](#_ENREF_26) |
| *NUDT3* | nudix hydrolase 3 | 6p21.31 | ADHD | [^47^](#_ENREF_47) |
| *NXPH1* | neurexophilin 1 | 7p21.3 | ADHD | [^29^](#_ENREF_29) |
| *NYAP2* | neuronal tyrosine-phosphorylated phosphoinositide-3-kinase adaptor 2 | 2q36.3 | ADHD | [^35^](#_ENREF_35) |
| *OPRM1* | opioid receptor mu 1 | 6q25.2 | ADHD | [^155^](#_ENREF_155) |
| *OR4C3* | olfactory receptor family 4 subfamily C member 3 | 11p11.2 | ADHD | [^3^](#_ENREF_3) |
| *OTOL1* | otolin 1 | 3q26.1 | ADHD |  |
| *OXER1* | oxoeicosanoid receptor 1 | 2p21 | ADHD | [^53^](#_ENREF_53) |
| *OXTR* | oxytocin receptor | 3p25.3 | ADHD | [^156^](#_ENREF_156) |
| *PARD3B* | par-3 family cell polarity regulator beta | 2q33.3 | ADHD | [^43^](#_ENREF_43) |
| *PARK2* | parkin RBR E3 ubiquitin protein ligase | 6q26 | ADHD | [^75^](#_ENREF_75) |
| *PER1* | period circadian clock 1 | 17p13.1 | ADHD | [^12^](#_ENREF_12) |
| *PER2* | period circadian clock 2 | 2q37.3 | ADHD | [^12^](#_ENREF_12) |
| *PEX5L* | peroxisomal biogenesis factor 5 like | 3q26.33 | ADHD | [^40^](#_ENREF_40) |
| *PGRMC2* | progesterone receptor membrane component 2 | 4q28.2 | ADHD | [^3^](#_ENREF_3) |
| *PHLDA1* | pleckstrin homology like domain family A member 1 | 12q21.2 | ADHD | [^3^](#_ENREF_3) |
| *PICK1* | protein interacting with PRKCA 1 | 22q13.1 | ADHD | [^35^](#_ENREF_35) |
| *PLCL1* | phospholipase C like 1 | 2q33.1 | ADHD | [^40^](#_ENREF_40) |
| *PNMT* | phenylethanolamine N-methyltransferase | 17q12 | ADHD |  |
| *POC5* | POC5 centriolar protein | 5q13.3 | ADHD | [^47^](#_ENREF_47) |
| *PPM1F* | protein phosphatase, Mg2+/Mn2+ dependent 1F | 22q11.22 | ADHD | [^27^](#_ENREF_27) |
| *PPM1H* | protein phosphatase, Mg2+/Mn2+ dependent 1H | 12q14.1-q14.2 | ADHD | [^29^](#_ENREF_29) |
| *PPP1R1B* | protein phosphatase 1 regulatory inhibitor subunit 1B | 17q12 | ADHD | [^157^](#_ENREF_157) |
| *PPP2R2C* | protein phosphatase 2 regulatory subunit Bgamma | 4p16.1 | ADHD | [^158^](#_ENREF_158) |
| *PRELID2* | PRELI domain containing 2 | 5q32 | ADHD | [^3^](#_ENREF_3) |
| *PRKAG2* | protein kinase AMP-activated non-catalytic subunit gamma 2 | 7q36.1 | ADHD | [^26^](#_ENREF_26) |
| *PRKD1* | protein kinase D1 | 14q12 | ADHD | [^47^](#_ENREF_47) |
| *PRKG1* | protein kinase, cGMP-dependent, type I | 10q11.23-q21.1 | ADHD |  |
| *PRTG* | protogenin | 15q21.3 | ADHD | [^102^](#_ENREF_102) |
| *PSMC3* | proteasome 26S subunit, ATPase 3 | 11p11.2 | ADHD | [^3^](#_ENREF_3) |
| *PTBP2* | polypyrimidine tract binding protein 2 | 1p21.3 | ADHD | [^47^](#_ENREF_47) |
| *PTCH1* | patched 1 | 9q22.32 | ADHD |  |
| *PTHLH* | parathyroid hormone like hormone | 12p11.22 | ADHD | [^53^](#_ENREF_53) |
| *PTPRG* | protein tyrosine phosphatase, receptor type G | 3p14.2 | ADHD | [^26^](#_ENREF_26) |
| *PTPRJ* | protein tyrosine phosphatase, receptor type J | 11p11.2 | ADHD | [^3^](#_ENREF_3) |
| *PTPRN2* | protein tyrosine phosphatase, receptor type N2 | 7q36.3 | ADHD | [^38^](#_ENREF_38) |
| *PYDC2* | pyrin domain containing 2 | 3q28 | ADHD | [^30^](#_ENREF_30) |
| *QPCTL* | glutaminyl-peptide cyclotransferase like | 19q13.32 | ADHD | [^47^](#_ENREF_47) |
| *RAB3A* | RAB3A, member RAS oncogene family | 19p13.11 | ADHD | [^74^](#_ENREF_74) |
| *RASSF2* | Ras association domain family member 2 | 20p13 | ADHD | [^35^](#_ENREF_35) |
| *RBMS3* | RNA binding motif single stranded interacting protein 3 | 3p24.1 | ADHD | [^34^](#_ENREF_34) |
| *RDH10* | retinol dehydrogenase 10 (all-trans) | 8q21.11 | ADHD | [^53^](#_ENREF_53) |
| *REEP5* | receptor accessory protein 5 | 5q22.2 | ADHD | [^27^](#_ENREF_27) |
| *RGS18* | regulator of G-protein signaling 18 | 1q31.2 | ADHD | [^3^](#_ENREF_3) |
| *RHOC* | ras homolog family member C | 1p13.2 | ADHD | [^53^](#_ENREF_53) |
| *RNF144B* | ring finger protein 144B | 6p22.3 | ADHD | [^53^](#_ENREF_53) |
| *RPL23AP56* | ribosomal protein L23a pseudogene 56 | 8q24.22 | ADHD | [^34^](#_ENREF_34) |
| *RPL27A* | ribosomal protein L27a | 11p15.4 | ADHD | [^47^](#_ENREF_47) |
| *RPL31P43* | ribosomal protein L31 pseudogene 43 | 9q31.2 | ADHD | [^34^](#_ENREF_34) |
| *RPL7P59* | ribosomal protein L7 pseudogene 59 | 7q35 | ADHD | [^34^](#_ENREF_34) |
| *SDK2* | sidekick cell adhesion molecule 2 | 17q25.1 | ADHD | [^27^](#_ENREF_27) |
| *SEC16B* | SEC16 homolog B, endoplasmic reticulum export factor | 1q25.2 | ADHD | [^47^](#_ENREF_47) |
| *SGTB* | small glutamine rich tetratricopeptide repeat containing beta | 5q12.3 | ADHD | [^67^](#_ENREF_67) |
| *SH2B1* | SH2B adaptor protein 1 | 16p11.2 | ADHD | [^47^](#_ENREF_47) |
| *SH3BP5* | SH3 domain binding protein 5 | 3p25.1 | ADHD | [^26^](#_ENREF_26) |
| *SHFM1* | split hand/foot malformation (ectrodactyly) type 1 | 7q21.3 | ADHD | [^26^](#_ENREF_26) |
| *SLC18A2* | solute carrier family 18 member A2 | 10q25.3 | ADHD |  |
| *SLC1A3* | solute carrier family 1 member 3 | 5p13.2 | ADHD |  |
| *SLC24A3* | solute carrier family 24 member 3 | 20p11.23 | MPH response | [^162^](#_ENREF_162) |
| *SLC38A1* | solute carrier family 38 member 1 | 12q13.11 | ADHD | [^35^](#_ENREF_35) |
| *SLC39A3* | solute carrier family 39 member 3 | 19p13.3 | ADHD | [^64^](#_ENREF_64) |
| *SLC39A8* | solute carrier family 39 member 8 | 4q24 | ADHD | [^47^](#_ENREF_47) |
| *SLC5A7* | solute carrier family 5 member 7 | 2q12.3 | ADHD | [^163^](#_ENREF_163) |
| *SLC6A1* | solute carrier family 6 member 1 | 3p25.3 | ADHD | [^12^](#_ENREF_12) |
| *SLC6A2* | solute carrier family 6 member 2 | 16q12.2 | ADHD and MPH response | ; For a review of ADHD genetic studies, see [^21^](#_ENREF_21) |
| *SLC6A3* | solute carrier family 6 member 3 | 5p15.33 | ADHD and MPH response | For a review of ADHD genetic and pharmacogenetic studies, see [^21^](#_ENREF_21) and , respectively |
| *SLC6A4* | solute carrier family 6 member 4 | 17q11.2 | ADHD and MPH response | ; For a review of ADHD genetic studies, see [^21^](#_ENREF_21) |
| *SLC7A10* | solute carrier family 7 member 10 | 19q13.11 | ADHD | [^67^](#_ENREF_67) |
| *SLC9A9* | solute carrier family 9 member A9 | 3q24 | ADHD |  |
| *SLCO3A1* | solute carrier organic anion transporter family member 3A1 | 15q26.1 | ADHD | [^53^](#_ENREF_53) |
| *SLCO5A1* | solute carrier organic anion transporter family member 5A1 | 8q13.3 | ADHD | [^26^](#_ENREF_26) |
| *SLIT1* | slit guidance ligand 1 | 10q24.1 | ADHD | [^40^](#_ENREF_40) |
| *SNAP23* | synaptosome associated protein 23 | 15q15.1-q15.2 | ADHD | [^46^](#_ENREF_46) |
| *SNAP25* | synaptosome associated protein 25 | 20p12.2 | ADHD and MPH response | ; For a review of ADHD genetic studies, see [^21^](#_ENREF_21) |
| *SNCA* | synuclein alpha | 4q22.1 | ADHD | [^3^](#_ENREF_3) |
| *SNPH* | syntaphilin | 20p13 | ADHD | [^74^](#_ENREF_74) |
| *SORCS1* | sortilin related VPS10 domain containing receptor 1 | 10q25.1 | ADHD | [^38^](#_ENREF_38) |
| *SORCS3* | sortilin related VPS10 domain containing receptor 3 | 10q25.1 | ADHD | [^38^](#_ENREF_38) |
| *SPATA13* | spermatogenesis associated 13 | 13q12.12 | ADHD |  |
| *SPOCK3* | SPARC/osteonectin, cwcv and kazal like domains proteoglycan 3 | 4q32.3 | ADHD | [^172^](#_ENREF_172) |
| *SRGAP1* | SLIT-ROBO Rho GTPase activating protein 1 | 12q14.2 | ADHD | [^40^](#_ENREF_40) |
| *SSFA2* | sperm specific antigen 2 | 2q31.3 | ADHD | [^3^](#_ENREF_3) |
| *STS* | steroid sulfatase (microsomal), isozyme S | Xp22.31 | ADHD | [^173-175^](#_ENREF_173) |
| *STX1A* | syntaxin 1A | 7q11.23 | ADHD |  |
| *STXBP1* | syntaxin binding protein 1 | 9q34.11 | ADHD | [^74^](#_ENREF_74) |
| *SUPT3H* | SPT3 homolog, SAGA and STAGA complex component | 6p21.1 | ADHD | [^27^](#_ENREF_27) |
| *SYN3* | synapsin III | 22q12.3 | ADHD |  |
| *SYP* | synaptophysin | Xp11.23 | ADHD |  |
| *SYT1* | synaptotagmin 1 | 12q21.2 | ADHD |  |
| *SYT16* | synaptotagmin 16 | 14q23.2 | ADHD | [^53^](#_ENREF_53) |
| *SYT2* | synaptotagmin 2 | 1q32.1 | ADHD | [^74^](#_ENREF_74) |
| *TAAR3* | trace amine associated receptor 3 (gene/pseudogene) | 6q23.2 | ADHD | [^181^](#_ENREF_181) |
| *TACR1* | tachykinin receptor 1 | 2p12 | ADHD | [^182^](#_ENREF_182) |
| *TACR3* | tachykinin receptor 3 | 4q24 | ADHD | [^42^](#_ENREF_42) |
| *TAF2* | TATA-box binding protein associated factor 2 | 8q24.12 | ADHD | [^35^](#_ENREF_35) |
| *TCERG1L* | transcription elongation regulator 1 like | 10q26.3 | ADHD | [^29^](#_ENREF_29) |
| *TDO2* | tryptophan 2,3-dioxygenase | 4q32.1 | ADHD | [^183^](#_ENREF_183) |
| *TDP2* | tyrosyl-DNA phosphodiesterase 2 | 6p22.3 | ADHD | [^76^](#_ENREF_76) |
| *TENM4* | teneurin transmembrane protein 4 | 11q14.1 | ADHD | [^30^](#_ENREF_30) |
| *TEX41* | testis expressed 41 (non-protein coding) | 2q22.3 | ADHD |  |
| *TFAP2B* | transcription factor AP-2 beta | 6p12.3 | ADHD | [^47^](#_ENREF_47) |
| *TFEB* | transcription factor EB | 6p21.1 | ADHD | [^27^](#_ENREF_27) |
| *TGFB2* | transforming growth factor beta 2 | 1q41 | ADHD | [^53^](#_ENREF_53) |
| *TH* | tyrosine hydroxylase | 11p15.5 | ADHD and MPH response |  |
| *TIAM2* | T-cell lymphoma invasion and metastasis 2 | 6q25.2-q25.3 | ADHD | [^40^](#_ENREF_40) |
| *TLE1* | transducin like enhancer of split 1 | 9q21.32 | ADHD | [^30^](#_ENREF_30) |
| *TLE4* | transducin like enhancer of split 4 | 9q21.31 | ADHD |  |
| *TLL1* | tolloid like 1 | 4q32.3 | ADHD |  |
| *TLL2* | tolloid like 2 | 10q24.1 | ADHD | [^27^](#_ENREF_27) |
| *TMEM132B* | transmembrane protein 132B | 12q24.31-q24.32 | ADHD | [^35^](#_ENREF_35) |
| *TMEM160* | transmembrane protein 160 | 19q13.32 | ADHD | [^47^](#_ENREF_47) |
| *TMEM18* | transmembrane protein 18 | 2p25.3 | ADHD | [^47^](#_ENREF_47) |
| *TMX3* | thioredoxin related transmembrane protein 3 | 18q22.1 | ADHD | [^35^](#_ENREF_35) |
| *TNFRSF10D* | TNF receptor superfamily member 10d | 8p21.3 | ADHD | [^26^](#_ENREF_26) |
| *TNNI3K* | TNNI3 interacting kinase | 1p31.1 | ADHD | [^47^](#_ENREF_47) |
| *TPH1* | tryptophan hydroxylase 1 | 11p15.1 | ADHD |  |
| *TPH2* | tryptophan hydroxylase 2 | 12q21.1 | ADHD and MPH response | ; For a review of ADHD genetic studies, see [^21^](#_ENREF_21) |
| *TRANK1* | tetratricopeptide repeat and ankyrin repeat containing 1 | 3p22.2 | ADHD | [^30^](#_ENREF_30) |
| *TRIM32* | tripartite motif containing 32 | 9q33.1 | ADHD | [^38^](#_ENREF_38) |
| *TRIO* | trio Rho guanine nucleotide exchange factor | 5p15.2 | ADHD | [^40^](#_ENREF_40) |
| *TRIQK* | triple QxxK/R motif containing | 8q22.1 | ADHD | [^26^](#_ENREF_26) |
| *TSHZ2* | teashirt zinc finger homeobox 2 | 20q13.2 | ADHD | [^26^](#_ENREF_26) |
| *TSPAN8* | tetraspanin 8 | 12q21.1 | ADHD | [^31^](#_ENREF_31) |
| *TTC12* | tetratricopeptide repeat domain 12 | 11q23.2 | ADHD |  |
| *UGT1A9* | UDP glucuronosyltransferase family 1 member A9 | 2q37.1 | ADHD | [^34^](#_ENREF_34) |
| *UNC5B* | unc-5 netrin receptor B | 10q22.1 | ADHD | [^27^](#_ENREF_27) |
| *USP24* | ubiquitin specific peptidase 24 | 1p32.3 | ADHD | [^67^](#_ENREF_67) |
| *VAMP1* | vesicle associated membrane protein 1 | 12p13.31 | ADHD | [^74^](#_ENREF_74) |
| *VAMP2* | vesicle associated membrane protein 2 | 17p13.1 | ADHD |  |
| *VEGFA* | vascular endothelial growth factor A | 6p21.1 | ADHD | [^3^](#_ENREF_3) |
| *WHRN* | whirlin | 9q32 | ADHD |  |
| *XKR3* | XK related 3 | 22q11.1 | ADHD | [^40^](#_ENREF_40) |
| *XKR4* | XK related 4 | 8q12.1 | ADHD | [^50^](#_ENREF_50) |
| *XPO1* | exportin 1 | 2p15 | ADHD | [^40^](#_ENREF_40) |
| *ZBBX* | zinc finger B-box domain containing | 3q26.1 | ADHD | [^38^](#_ENREF_38) |
| *ZMAT4* | zinc finger matrin-type 4 | 8p11.21 | ADHD | [^53^](#_ENREF_53) |
| *ZNF385D* | zinc finger protein 385D | 3p24.3 | ADHD | [^53^](#_ENREF_53) |
| *ZNF423* | zinc finger protein 423 | 16q12.1 | ADHD | [^53^](#_ENREF_53) |
| *ZNF516* | zinc finger protein 516 | 18q23 | ADHD | [^35^](#_ENREF_35) |
| *ZNF544* | zinc finger protein 544 | 19q13.43 | ADHD | [^53^](#_ENREF_53) |
| *ZNF608* | zinc finger protein 608 | 5q23.2 | ADHD | [^47^](#_ENREF_47) |
| *ZNF75A* | zinc finger protein 75a | 16p13.3 | ADHD | [^26^](#_ENREF_26) |
| *ZNF804A* | zinc finger protein 804A | 2q32.1 | ADHD |  |
| *ZNF805* | zinc finger protein 805 | 19q13.43 | ADHD | [^53^](#_ENREF_53) |

Note: ADHD, attention-deficit/hyperactivity disorder; MPH, methylphenidate.

^a, b, c^All relative to the human reference genome GRCh38 (NCBI Build 38).

**Supplementary Table S2** Demographic and clinical characteristics of the discovery population in the GWAS analysis according to methylphenidate response^a^

|  | Non-responders, N (%) 32 (18.5) | Responders, N (%) 141 (81.5) | P-value^b^ |
| --- | --- | --- | --- |
| Age, mean (SD) | 10.0 (2.98) | 9.50 (2.89) | 0.382 |
| Sex, N (%) |  |  | 0.998 |
| Male | 27 (84.4) | 119 (84.4) |  |
| Female | 5 (15.6) | 22 (15.6) |  |
| ADHD subtype, N (%) |  |  | 0.540 |
| Combined-type | 27 (84.4) | 104 (73.8) |  |
| Inattentive type | 5 (15.6) | 32 (22.7) |  |
| Hyperactive-impulsive type | 0 (0) | 5 (3.5) |  |
| Comorbid conditions, N (%) | 7 (21.9) | 32 (22.7) | 0.920 |
| Disabilities in reading and writing | 4 (12.5) | 18 (12.8) | 0.967 |
| ODD | 3 (9.4) | 7 (5.0) | 0.334 |
| Anxiety disorders | 0 (0) | 2 (1.4) | 1.000 |
| Dyslexia | 0 (0) | 1 (0.7) | 1.000 |
| Tic disorders | 0 (0) | 3 (2.1) | 1.000 |
| Neurodevelopmental disorders | 0 (0) | 1 (0.7) | 1.000 |
| CGI-S baseline score, mean (SD) | 5.19 (1.06) | 4.77 (0.71) | 0.001 |
| Use of concomitant medication, N (%) | 1 (3.1) | 6 (4.3) | 0.770 |
| MPH dose prescribed (mg/kg), mean (SD) | 0.99 (0.28) | 1.08 (0.27) | 0.107 |
| Drug formulation, N (%) |  |  | 0.124 |
| Immediate-release MPH | 11 (34.4) | 26 (18.4) |  |
| Extended-release MPH | 20 (62.5) | 110 (78.0) |  |
| Long-acting MPH | 1 (3.1) | 5 (3.5) |  |

GWAS, genome-wide association study; SD, standard deviation; ADHD, attention-deficit/hyperactivity disorder; ODD, oppositional defiant disorder; CGI-S, Clinical Global Impression-Severity scale; MPH, methylphenidate.

^a^Clinical response to MPH defined by a Clinical Global Impression-Improvement score of two points or less.

^b^Calculated using either Pearson’s Chi-square test or Fisher’s exact test for categorical variables and Student’s *t*-test for continuous variables.

**Supplementary Table S5** Demographic and clinical characteristics of the target population used both in PRS analysis and meta-analysis according to methylphenidate response^a^

|  | Non-responders, N (%) 28 (14.8) | | Responders, N (%) 161 (85.2) | | P-value^b^ | |  |
| --- | --- | --- | --- | --- | --- | --- | --- |
| Age, mean (SD) | 34.4 (11.2) | | 33.4 (10.6) | | 0.664 | |  |
| Sex, N (%) |  | |  | | 0.964 | |  |
| Male | 15 (53.6) | | 87 (54.0) | |  | |  |
| Female | 13 (46.4) | | 74 (46.0) | |  | |  |
| ADHD subtype, N (%) |  | |  | | 0.097 | |  |
| Combined-type | 11 (39.3) | | 97 (60.2) | |  | |  |
| Inattentive type | 16 (57.1) | | 62 (38.5) | |  | |  |
| Hyperactive-impulsive type | 1 (3.6) | | 2 (1.2) | |  | |  |
| Comorbid conditions, N (%) | 24 (85.7) | | 152 (94.4) | | 0.093 | |  |
| ODD | 10 (35.7) | | 87 (54.0) | | 0.054 | |  |
| Anxiety disorders^c^ | 6 (21.4) | | 39 (24.2) | | 0.749 | |  |
| Phobia | 13 (46.4) | | 34 (21.1) | | 0.004 | |  |
| Mood disorders^d^ | 15 (53.6) | | 87 (54.0) | | 0.964 | |  |
| SUD | 11 (39.3) | | 79 (49.1) | | 0.339 | |  |
| CGI-S baseline score, mean (SD) | 4.32 (0.67) | | 4.66 (0.73) | | 0.024 | |  |
| Use of concomitant medication, N (%) | 16 (57.1) | | 46 (28.6) | | 0.003 | |  |
| MPH dose prescribed (mg/kg), mean (SD)^e^ | | 0.53 (0.20) | | 0.54 (0.20) | | 0.916 | |

PRS, polygenic risk score; SD, standard deviation; ADHD, attention-deficit/hyperactivity disorder; ODD, oppositional defiant disorder; SUD, substance use disorder; CGI-S, Clinical Global Impression-Severity scale; MPH, methylphenidate.

^a^Clinical response to MPH defined by a Clinical Global Impression-Improvement score of two points or less.

^b^Calculated using either Pearson’s Chi-square test or Fisher’s exact test for categorical variables and Student’s *t*-test for continuous variables.

^c^Includes generalised anxiety disorder and panic disorder.

^d^Includes major depressive disorder and bipolar disorder.

^e^All individuals received immediate-release MPH.

**References**

1. Chaste, P. *et al*. Genetic variations of the melatonin pathway in patients with attention-deficit and hyperactivity disorders. *J Pineal Res*. **51,** 394-399 (2011).

2. Kim, S. W. *et al*. ABCB1 c.2677G>T variation is associated with adverse reactions of OROS-methylphenidate in children and adolescents with ADHD. *J Clin Psychopharmacol*. **33,** 491-498 (2013).

3. Hinney, A. *et al*. Genome-wide association study in German patients with attention deficit/hyperactivity disorder. *Am J Med Genet B Neuropsychiatr Genet*. **156B,** 888-897 (2011).

4. Bruxel, E. M. *et al*. LPHN3 and attention-deficit/hyperactivity disorder: a susceptibility and pharmacogenetic study. *Genes Brain Behav*. **14,** 419-427 (2015).

5. Song, J. *et al*. Association of SNAP-25, SLC6A2, and LPHN3 with OROS methylphenidate treatment response in attention-deficit/hyperactivity disorder. *Clin Neuropharmacol*. **37,** 136-141 (2014).

6. Jain, M. *et al*. A cooperative interaction between LPHN3 and 11q doubles the risk for ADHD. *Mol Psychiatry*. **17,** 741-747 (2012).

7. Labbe, A. *et al*. Refining psychiatric phenotypes for response to treatment: contribution of LPHN3 in ADHD. *Am J Med Genet B Neuropsychiatr Genet*. **159B,** 776-785 (2012).

8. Arcos-Burgos, M. *et al*. A common variant of the latrophilin 3 gene, LPHN3, confers susceptibility to ADHD and predicts effectiveness of stimulant medication. *Mol Psychiatry*. **15,** 1053-1066 (2010).

9. Domené, S. *et al*. Screening of human LPHN3 for variants with a potential impact on ADHD susceptibility. *Am J Med Genet B Neuropsychiatr Genet*. **156B,** 11-18 (2011).

10. Ribasés, M. *et al*. Contribution of LPHN3 to the genetic susceptibility to ADHD in adulthood: a replication study. *Genes Brain Behav*. **10,** 149-157 (2011).

11. Barr, C. L. *et al*. Attention-deficit hyperactivity disorder and the adrenergic receptors alpha 1C and alpha 2C. *Mol Psychiatry*. **6,** 334-337 (2001).

12. Brookes, K. *et al*. The analysis of 51 genes in DSM-IV combined type attention deficit hyperactivity disorder: association signals in DRD4, DAT1 and 16 other genes. *Mol Psychiatry*. **11,** 934-953 (2006).

13. Hawi, Z. *et al*. A high density linkage disequilibrium mapping in 14 noradrenergic genes: evidence of association between SLC6A2, ADRA1B and ADHD. *Psychopharmacology (Berl)*. **225,** 895-902 (2013).

14. Kim, B. N. *et al*. Norepinephrine genes predict response time variability and methylphenidate-induced changes in neuropsychological function in attention deficit hyperactivity disorder. *J Clin Psychopharmacol*. **33,** 356-362 (2013).

15. Hong, S. B. *et al*. Dopaminergic and noradrenergic gene polymorphisms and response to methylphenidate in korean children with attention-deficit/hyperactivity disorder: is there an interaction? *J Child Adolesc Psychopharmacol*. **22,** 343-352 (2012).

16. Froehlich, T. E. *et al*. Pharmacogenetic predictors of methylphenidate dose-response in attention-deficit/hyperactivity disorder. *J Am Acad Child Adolesc Psychiatry*. **50,** 1129-1139 e1122 (2011).

17. Contini, V. *et al*. Adrenergic alpha2A receptor gene is not associated with methylphenidate response in adults with ADHD. *Eur Arch Psychiatry Clin Neurosci*. **261,** 205-211 (2011).

18. Cheon, K. A., Cho, D. Y., Koo, M. S., Song, D. H. & Namkoong, K. Association between homozygosity of a G allele of the alpha-2a-adrenergic receptor gene and methylphenidate response in Korean children and adolescents with attention-deficit/hyperactivity disorder. *Biol Psychiatry*. **65,** 564-570 (2009).

19. da Silva, T. L. *et al*. Adrenergic alpha2A receptor gene and response to methylphenidate in attention-deficit/hyperactivity disorder-predominantly inattentive type. *J Neural Transm (Vienna)*. **115,** 341-345 (2008).

20. Polanczyk, G. *et al*. Association of the adrenergic alpha2A receptor gene with methylphenidate improvement of inattentive symptoms in children and adolescents with attention-deficit/hyperactivity disorder. *Arch Gen Psychiatry*. **64,** 218-224 (2007).

21. Li, Z., Chang, S. H., Zhang, L. Y., Gao, L. & Wang, J. Molecular genetic studies of ADHD and its candidate genes: a review. *Psychiatry Res*. **219,** 10-24 (2014).

22. Guan, L. *et al*. A high-density single-nucleotide polymorphism screen of 23 candidate genes in attention deficit hyperactivity disorder: suggesting multiple susceptibility genes among Chinese Han population. *Mol Psychiatry*. **14,** 546-554 (2009).

23. Cho, S. C. *et al*. Association between the alpha-2C-adrenergic receptor gene and attention deficit hyperactivity disorder in a Korean sample. *Neurosci Lett*. **446,** 108-111 (2008).

24. De Luca, V. *et al*. Adrenergic alpha 2C receptor genomic organization: association study in adult ADHD. *Am J Med Genet B Neuropsychiatr Genet*. **127B,** 65-67 (2004).

25. Feng, J. *et al*. An in-frame deletion in the alpha(2C) adrenergic receptor is common in African--Americans. *Mol Psychiatry*. **6,** 168-172 (2001).

26. Neale, B. M. *et al*. Meta-analysis of genome-wide association studies of attention-deficit/hyperactivity disorder. *J Am Acad Child Adolesc Psychiatry*. **49,** 884-897 (2010).

27. Lesch, K. P. *et al*. Molecular genetics of adult ADHD: converging evidence from genome-wide association and extended pedigree linkage studies. *J Neural Transm (Vienna)*. **115,** 1573-1585 (2008).

28. Lyon, G. J. *et al*. Exome sequencing and unrelated findings in the context of complex disease research: ethical and clinical implications. *Discov Med*. **12,** 41-55 (2011).

29. Neale, B. M. *et al*. Case-control genome-wide association study of attention-deficit/hyperactivity disorder. *J Am Acad Child Adolesc Psychiatry*. **49,** 906-920 (2010).

30. Schimmelmann, B. G. *et al*. Bipolar disorder risk alleles in children with ADHD. *J Neural Transm (Vienna)*. **120,** 1611-1617 (2013).

31. Landaas, E. T. *et al*. Bipolar disorder risk alleles in adult ADHD patients. *Genes Brain Behav*. **10,** 418-423 (2011).

32. Mota, N. R. *et al*. NCAM1-TTC12-ANKK1-DRD2 gene cluster and the clinical and genetic heterogeneity of adults with ADHD. *Am J Med Genet B Neuropsychiatr Genet*. **168,** 433-444 (2015).

33. Nyman, E. S. *et al*. ADHD candidate gene study in a population-based birth cohort: association with DBH and DRD2. *J Am Acad Child Adolesc Psychiatry*. **46,** 1614-1621 (2007).

34. Mick, E. *et al*. Family-based genome-wide association scan of attention-deficit/hyperactivity disorder. *J Am Acad Child Adolesc Psychiatry*. **49,** 898-905 e893 (2010).

35. Yang, L. *et al*. Polygenic transmission and complex neuro developmental network for attention deficit hyperactivity disorder: genome-wide association study of both common and rare variants. *Am J Med Genet B Neuropsychiatr Genet*. **162B,** 419-430 (2013).

36. Michaelovsky, E. *et al*. Association between a common haplotype in the COMT gene region and psychiatric disorders in individuals with 22q11.2DS. *Int J Neuropsychopharmacol*. **11,** 351-363 (2008).

37. Cross-Disorder Group of the Psychiatric Genomics Consortium. Identification of risk loci with shared effects on five major psychiatric disorders: a genome-wide analysis. *Lancet*. **381,** 1371-1379 (2013).

38. Lionel, A. C. *et al*. Rare copy number variation discovery and cross-disorder comparisons identify risk genes for ADHD. *Sci Transl Med*. **3,** 95ra75 (2011).

39. Ribasés, M. *et al*. Case-control study of six genes asymmetrically expressed in the two cerebral hemispheres: association of BAIAP2 with attention-deficit/hyperactivity disorder. *Biol Psychiatry*. **66,** 926-934 (2009).

40. Stergiakouli, E. *et al*. Investigating the contribution of common genetic variants to the risk and pathogenesis of ADHD. *Am J Psychiatry*. **169,** 186-194 (2012).

41. Williams, N. M. *et al*. Rare chromosomal deletions and duplications in attention-deficit hyperactivity disorder: a genome-wide analysis. *Lancet*. **376,** 1401-1408 (2010).

42. Elia, J. *et al*. Rare structural variants found in attention-deficit hyperactivity disorder are preferentially associated with neurodevelopmental genes. *Mol Psychiatry*. **15,** 637-646 (2010).

43. Ebejer, J. L. *et al*. Genome-wide association study of inattention and hyperactivity-impulsivity measured as quantitative traits. *Twin Res Hum Genet*. **16,** 560-574 (2013).

44. Jacob, C. P. *et al*. Acetylcholine-metabolizing butyrylcholinesterase (BCHE) copy number and single nucleotide polymorphisms and their role in attention-deficit/hyperactivity syndrome. *J Psychiatr Res*. **47,** 1902-1908 (2013).

45. Kim, B. N. *et al*. Val/Val genotype of brain-derived neurotrophic factor (BDNF) Val(6)(6)Met polymorphism is associated with a better response to OROS-MPH in Korean ADHD children. *Int J Neuropsychopharmacol*. **14,** 1399-1410 (2011).

46. Zhang, L. *et al*. ADHDgene: a genetic database for attention deficit hyperactivity disorder. *Nucleic Acids Res*. **40,** D1003-1009 (2012).

47. Albayrak, O. *et al*. Common obesity risk alleles in childhood attention-deficit/hyperactivity disorder. *Am J Med Genet B Neuropsychiatr Genet*. **162B,** 295-305 (2013).

48. Laurin, N. *et al*. Association of the calcyon gene (DRD1IP) with attention deficit/hyperactivity disorder. *Mol Psychiatry*. **10,** 1117-1125 (2005).

49. Hsu, C. D. Family-based association study of cocaine- and amphetamine-regulated transcript (CARTPT) and protein interaction with C-kinase-1 (PICK1) genes in attention-deficit hyperactivity disorder. *Psychiatry Res*. **198,** 334-335 (2012).

50. Lantieri, F., Glessner, J. T., Hakonarson, H., Elia, J. & Devoto, M. Analysis of GWAS top hits in ADHD suggests association to two polymorphisms located in genes expressed in the cerebellum. *Am J Med Genet B Neuropsychiatr Genet*. **153B**(6)**,** 1127-1133 (2010).

51. Salatino-Oliveira, A. *et al*. Cadherin-13 gene is associated with hyperactive/impulsive symptoms in attention/deficit hyperactivity disorder. *Am J Med Genet B Neuropsychiatr Genet*. **168B,** 162-169 (2015).

52. Mavroconstanti, T., Johansson, S., Winge, I., Knappskog, P. M. & Haavik, J. Functional properties of rare missense variants of human CDH13 found in adult attention deficit/hyperactivity disorder (ADHD) patients. *PLoS One*. **8,** e71445 (2013).

53. Lasky-Su, J. *et al*. Genome-wide association scan of quantitative traits for attention deficit hyperactivity disorder identifies novel associations and confirms candidate gene associations. *Am J Med Genet B Neuropsychiatr Genet*. **147B,** 1345-1354 (2008).

54. Nemoda, Z., Angyal, N., Tarnok, Z., Gadoros, J. & Sasvari-Szekely, M. Carboxylesterase 1 gene polymorphism and methylphenidate response in ADHD. *Neuropharmacology*. **57,** 731-733 (2009).

55. Thakur, G. A., Sengupta, S. M., Grizenko, N., Choudhry, Z. & Joober, R. Family-based association study of ADHD and genes increasing the risk for smoking behaviours. *Arch Dis Child*. **97,** 1027-1033 (2012).

56. Lee, J. *et al*. Association study of the nicotinic acetylcholine receptor alpha4 subunit gene, CHRNA4, in attention-deficit hyperactivity disorder. *Genes Brain Behav*. **7,** 53-60 (2008).

57. Tharoor, H., Lobos, E. A., Todd, R. D. & Reiersen, A. M. Association of dopamine, serotonin, and nicotinic gene polymorphisms with methylphenidate response in ADHD. *Am J Med Genet B Neuropsychiatr Genet*. **147B,** 527-530 (2008).

58. Bobb, A. J. *et al*. Support for association between ADHD and two candidate genes: NET1 and DRD1. *Am J Med Genet B Neuropsychiatr Genet*. **134B,** 67-72 (2005).

59. Todd, R. D., Lobos, E. A., Sun, L. W. & Neuman, R. J. Mutational analysis of the nicotinic acetylcholine receptor alpha 4 subunit gene in attention deficit/hyperactivity disorder: evidence for association of an intronic polymorphism with attention problems. *Mol Psychiatry*. **8,** 103-108 (2003).

60. Kent, L. *et al*. Nicotinic acetylcholine receptor alpha4 subunit gene polymorphism and attention deficit hyperactivity disorder. *Psychiatr Genet*. **11,** 37-40 (2001).

61. Kent, L. *et al*. No association between CHRNA7 microsatellite markers and attention-deficit hyperactivity disorder. *Am J Med Genet*. **105,** 686-689 (2001).

62. Xu, X. *et al*. Association study between a polymorphism at the 3'-untranslated region of CLOCK gene and attention deficit hyperactivity disorder. *Behav Brain Funct*. **6,** 48 (2010).

63. Kissling, C. *et al*. A polymorphism at the 3'-untranslated region of the CLOCK gene is associated with adult attention-deficit hyperactivity disorder. *Am J Med Genet B Neuropsychiatr Genet*. **147,** 333-338 (2008).

64. Weber, H. *et al*. Cross-disorder analysis of bipolar risk genes: further evidence of DGKH as a risk gene for bipolar disorder, but also unipolar depression and adult ADHD. *Neuropsychopharmacology*. **36,** 2076-2085 (2011).

65. Lu, A. T. *et al*. Association of the cannabinoid receptor gene (CNR1) with ADHD and post-traumatic stress disorder. *Am J Med Genet B Neuropsychiatr Genet*. **147B,** 1488-1494 (2008).

66. Ribasés, M. *et al*. Association study of 10 genes encoding neurotrophic factors and their receptors in adult and child attention-deficit/hyperactivity disorder. *Biol Psychiatry*. **63,** 935-945 (2008).

67. Elia, J. *et al*. Genome-wide copy number variation study associates metabotropic glutamate receptor gene networks with attention deficit hyperactivity disorder. *Nat Genet*. **44,** 78-84 (2011).

68. Zhou, K. J *et al*. Meta-analysis of genome-wide linkage scans of attention deficit hyperactivity disorder. *Am J Med Genet B Neuropsychiatr Genet*. **147B,** 1392-1398 (2008).

69. Pagerols, M. *et al*. Pharmacogenetics of methylphenidate response and tolerability in attention-deficit/hyperactivity disorder. *Pharmacogenomics J*. **17,** 98-104 (2017).

70. Contini, V. *et al*. No significant association between genetic variants in 7 candidate genes and response to methylphenidate treatment in adult patients with ADHD. *J Clin Psychopharmacol*. **32,** 820-823 (2012).

71. McGough, J. J. *et al*. A candidate gene analysis of methylphenidate response in attention-deficit/hyperactivity disorder. *J Am Acad Child Adolesc Psychiatry*. **48,** 1155-1164 (2009).

72. Cheon, K. A., Jun, J. Y. & Cho, D. Y. Association of the catechol-O-methyltransferase polymorphism with methylphenidate response in a classroom setting in children with attention-deficit hyperactivity disorder. *Int Clin Psychopharmacol*. **23,** 291-298 (2008).

73. Kereszturi, E. *et al*. Catechol-O-methyltransferase Val158Met polymorphism is associated with methylphenidate response in ADHD children. *Am J Med Genet B Neuropsychiatr Genet*. **147B,** 1431-1435 (2008).

74. Sánchez-Mora, C. *et al*. Evaluation of common variants in 16 genes involved in the regulation of neurotransmitter release in ADHD. *Eur Neuropsychopharmacol*. **23,** 426-435 (2013).

75. Jarick, I. *et al*. Genome-wide analysis of rare copy number variations reveals PARK2 as a candidate gene for attention-deficit/hyperactivity disorder. *Mol Psychiatry*. **19,** 115-121 (2014).

76. Couto, J. M. *et al*. Association of attention-deficit/hyperactivity disorder with a candidate region for reading disabilities on chromosome 6p. *Biol Psychiatry*. **66,** 368-375 (2009).

77. Havik, B. *et al*. DCLK1 variants are associated across schizophrenia and attention deficit/hyperactivity disorder. *PLoS One*. **7,** e35424 (2012).

78. Ribasés, M. *et al*. Exploration of 19 serotoninergic candidate genes in adults and children with attention-deficit/hyperactivity disorder identifies association for 5HT2A, DDC and MAOB. *Mol Psychiatry*. **14,** 71-85 (2009).

79. Hawi, Z. *et al*. Dopa decarboxylase gene polymorphisms and attention deficit hyperactivity disorder (ADHD): no evidence for association in the Irish population. *Mol Psychiatry*. **6,** 420-424 (2001).

80. Hawi, Z. *et al*. Preferential transmission of paternal alleles at risk genes in attention-deficit/hyperactivity disorder. *Am J Hum Genet*. **77,** 958-965 (2005).

81. Kirley, A. *et al*. Dopaminergic system genes in ADHD: toward a biological hypothesis. *Neuropsychopharmacology*. **27,** 607-619 (2002).

82. Reif, A. *et al*. DIRAS2 is associated with adult ADHD, related traits, and co-morbid disorders. *Neuropsychopharmacology*. **36,** 2318-2327 (2011).

83. Jacobsen, K. K. *et al*. DISC1 in adult ADHD patients: an association study in two European samples. *Am J Med Genet B Neuropsychiatr Genet*. **162B,** 227-234 (2013).

84. Neale, B. M. *et al*. Genome-wide association scan of attention deficit hyperactivity disorder. *Am J Med Genet B Neuropsychiatr Genet*. **147B,** 1337-1344 (2008).

85. Ribasés, M. *et al*. Candidate system analysis in ADHD: evaluation of nine genes involved in dopaminergic neurotransmission identifies association with DRD1. *World J Biol Psychiatry*. **13,** 281-292 (2012).

86. Oades, R. D. *et al*. The influence of serotonin- and other genes on impulsive behavioral aggression and cognitive impulsivity in children with attention-deficit/hyperactivity disorder (ADHD): Findings from a family-based association test (FBAT) analysis. *Behav Brain Funct*. **4,** 48 (2008).

87. Wang, G. X., Ma, Y. H., Wang, S. F., Ren, G. F. & Guo, H. Association of dopaminergic/GABAergic genes with attention deficit hyperactivity disorder in children. *Mol Med Rep*. **6,** 1093-1098 (2012).

88. Misener, V. L. *et al*. Linkage of the dopamine receptor D1 gene to attention-deficit/hyperactivity disorder. *Mol Psychiatry*. **9,** 500-509 (2004).

89. Hasler, R. *et al*. DAT1 and DRD4 genes involved in key dimensions of adult ADHD. *Neurol Sci*. **36,** 861-869 (2015).

90. Qian, Q. *et al*. Evaluation of potential gene-gene interactions for attention deficit hyperactivity disorder in the Han Chinese population. *Am J Med Genet B Neuropsychiatr Genet*. **144B,** 200-206 (2007).

91. Huang, Y. S., Lin, S. K., Wu, Y. Y., Chao, C. & Chen, C. K. A family-based association study of attention-deficit hyperactivity disorder and dopamine D2 receptor TaqI A alleles. *Chang Gung Med J*. **26,** 897-903 (2003).

92. Kustanovich, V. *et al*. Transmission disequilibrium testing of dopamine-related candidate gene polymorphisms in ADHD: confirmation of association of ADHD with DRD4 and DRD5. *Mol Psychiatry*. **9,** 711-717 (2004).

93. Rowe, D. C. *et al*. The DRD2 TaqI polymorphism and symptoms of attention deficit hyperactivity disorder. *Mol Psychiatry*. **4,** 580-586 (1999).

94. Comings, D. E. *et al*. The dopamine D2 receptor locus as a modifying gene in neuropsychiatric disorders. *JAMA*. **266,** 1793-1800 (1991).

95. Winsberg, B. G. & Comings, D. E. Association of the dopamine transporter gene (DAT1) with poor methylphenidate response. *J Am Acad Child Adolesc Psychiatry*. **38,** 1474-1477 (1999).

96. Muglia, P., Jain, U. & Kennedy, J. L. A transmission disequilibrium test of the Ser9/Gly dopamine D3 receptor gene polymorphism in adult attention-deficit hyperactivity disorder. *Behav Brain Res*. **130,** 91-95 (2002).

97. Payton, A. *et al*. Examining for association between candidate gene polymorphisms in the dopamine pathway and attention-deficit hyperactivity disorder: a family-based study. *Am J Med Genet*. **105,** 464-470 (2001).

98. Barr, C. L. *et al*. Linkage study of two polymorphisms at the dopamine D3 receptor gene and attention-deficit hyperactivity disorder. *Am J Med Genet*. **96,** 114-117 (2000).

99. Bruxel, E. M. *et al*. ADHD pharmacogenetics across the life cycle: New findings and perspectives. *Am J Med Genet B Neuropsychiatr Genet*. **165B,** 263-282 (2014).

100. Froehlich, T. E., McGough, J. J. & Stein, M. A. Progress and promise of attention-deficit hyperactivity disorder pharmacogenetics. *CNS Drugs*. **24,** 99-117 (2010).

101. Tahir, E. *et al*. Association and linkage of DRD4 and DRD5 with attention deficit hyperactivity disorder (ADHD) in a sample of Turkish children. *Mol Psychiatry*. **5,** 396-404 (2000).

102. Wigg, K. G. *et al*. Association of ADHD and the Protogenin gene in the chromosome 15q21.3 reading disabilities linkage region. *Genes Brain Behav*. **7,** 877-886 (2008).

103. Brookes, K. J., Chen, W., Xu, X., Taylor, E. & Asherson, P. Association of fatty acid desaturase genes with attention-deficit/hyperactivity disorder. *Biol Psychiatry*. **60,** 1053-1061 (2006).

104. Sánchez-Mora, C. *et al*. Case-control genome-wide association study of persistent attention-deficit hyperactivity disorder identifies FBXO33 as a novel susceptibility gene for the disorder. *Neuropsychopharmacology*. **40,** 915-926 (2015).

105. Laurin, N. *et al*. Association study for genes at chromosome 5p13-q11 in attention deficit hyperactivity disorder. *Am J Med Genet B Neuropsychiatr Genet*. **147B,** 600-605 (2008).

106. Ribasés, M. *et al*. An association study of sequence variants in the forkhead box P2 (FOXP2) gene and adulthood attention-deficit/hyperactivity disorder in two European samples. *Psychiatr Genet*. **22,** 155-160 (2012).

107. Choudhry, Z. *et al*. Association between obesity-related gene FTO and ADHD. *Obesity (Silver Spring)*. **21,** E738-744 (2013).

108. Syed, Z., Dudbridge, F. & Kent, L. An investigation of the neurotrophic factor genes GDNF, NGF, and NT3 in susceptibility to ADHD. *Am J Med Genet B Neuropsychiatr Genet*. **144B,** 375-378 (2007).

109. Salatino-Oliveira, A. *et al*. Association study of GIT1 gene with attention-deficit hyperactivity disorder in Brazilian children and adolescents. *Genes Brain Behav*. **11,** 864-868 (2012).

110. Won, H. *et al*. GIT1 is associated with ADHD in humans and ADHD-like behaviors in mice. *Nat Med*. **17,** 566-572 (2011).

111. Laurin, N. *et al*. Investigation of the G protein subunit Galphaolf gene (GNAL) in attention deficit/hyperactivity disorder. *J Psychiatr Res*. **42,** 117-124 (2008).

112. Turic, D. *et al*. Direct analysis of the genes encoding G proteins G alpha T2, G alpha o, G alpha Z in ADHD. *Am J Med Genet B Neuropsychiatr Genet*. **127B,** 68-72 (2004).

113. Romanos, M. *et al*. Genome-wide linkage analysis of ADHD using high-density SNP arrays: novel loci at 5q13.1 and 14q12. *Mol Psychiatry*. **13,** 522-530 (2008).

114. Park, S. *et al*. Association between the GRM7 rs3792452 polymorphism and attention deficit hyperacitiveity disorder in a Korean sample. *Behav Brain Funct*. **9,** 1 (2013).

115. Adams, J. *et al*. Glutamate receptor, ionotropic, N-methyl D-aspartate 2A (GRIN2A) gene as a positional candidate for attention-deficit/hyperactivity disorder in the 16p13 region. *Mol Psychiatry*. **9,** 494-499 (2004).

116. Turic, D. *et al*. Follow-up of genetic linkage findings on chromosome 16p13: evidence of association of N-methyl-D aspartate glutamate receptor 2A gene polymorphism with ADHD. *Mol Psychiatry*. **9,** 169-173 (2004).

117. Dorval, K. M. *et al*. Association of the glutamate receptor subunit gene GRIN2B with attention-deficit/hyperactivity disorder. *Genes Brain Behav*. **6,** 444-452 (2007).

118. Mick, E., Neale, B., Middleton, F. A., McGough, J. J. & Faraone, S. V. Genome-wide association study of response to methylphenidate in 187 children with attention-deficit/hyperactivity disorder. *Am J Med Genet B Neuropsychiatr Genet*. **147B,** 1412-1418 (2008).

119. Shim, S. H. *et al*. Association between glycogen synthase kinase-3beta gene polymorphisms and attention deficit hyperactivity disorder in Korean children: a preliminary study. *Prog Neuropsychopharmacol Biol Psychiatry*. **39,** 57-61 (2012).

120. Arcos-Burgos, M. *et al*. Attention-deficit/hyperactivity disorder in a population isolate: linkage to loci at 4q13.2, 5q33.3, 11q22, and 17p11. *Am J Hum Genet*. **75,** 998-1014 (2004).

121. Hebebrand, J. *et al*. A genome-wide scan for attention-deficit/hyperactivity disorder in 155 German sib-pairs. *Mol Psychiatry*. **11,** 196-205 (2006).

122. Aureli, A. *et al*. Investigation on the possible relationship existing between the HLA-DR gene and attention deficit hyperactivity disorder and/or mental retardation. *Int J Immunopathol Pharmacol*. **21,** 985-991 (2008).

123. Payton, A. *et al*. No evidence of association between HLA-DRB1 and attention deficit hyperactivity disorder. *Psychiatr Genet*. **13,** 183-185 (2003).

124. Park, Y. H. *et al*. Association between HTR1A gene polymorphisms and attention deficit hyperactivity disorder in Korean children. *Genet Test Mol Biomarkers*. **17,** 178-182 (2013).

125. Shim, S. H. *et al*. A case-control association study of serotonin 1A receptor gene and tryptophan hydroxylase 2 gene in attention deficit hyperactivity disorder. *Prog Neuropsychopharmacol Biol Psychiatry*. **34,** 974-979 (2010).

126. Zeni, C. P. *et al*. No significant association between response to methylphenidate and genes of the dopaminergic and serotonergic systems in a sample of Brazilian children with attention-deficit/hyperactivity disorder. *Am J Med Genet B Neuropsychiatr Genet*. **144B,** 391-394 (2007).

127. Li, J. *et al*. The serotonin 5-HT1D receptor gene and attention-deficit hyperactivity disorder in Chinese Han subjects. *Am J Med Genet B Neuropsychiatr Genet*. **141B,** 874-876 (2006).

128. Xu, X., Brookes, K., Sun, B., Ilott, N. & Asherson, P. Investigation of the serotonin 2C receptor gene in attention deficit hyperactivity disorder in UK samples. *BMC Res Notes*. **2,** 71 (2009).

129. Li, J. *et al*. Association between polymorphisms in serotonin 2C receptor gene and attention-deficit/hyperactivity disorder in Han Chinese subjects. *Neurosci Lett*. **407,** 107-111 (2006).

130. Li, J. *et al*. Association of attention-deficit/hyperactivity disorder with serotonin 4 receptor gene polymorphisms in Han Chinese subjects. *Neurosci Lett*. **401,** 6-9 (2006).

131. Li, J. *et al*. No association of attention-deficit/hyperactivity disorder with genes of the serotonergic pathway in Han Chinese subjects. *Neurosci Lett*. **403,** 172-175 (2006).

132. Misener, V. L. *et al*. Replication test for association of the IL-1 receptor antagonist gene, IL1RN, with attention-deficit/hyperactivity disorder. *Neuropsychobiology*. **50,** 231-234 (2004).

133. Segman, R. H. *et al*. Preferential transmission of interleukin-1 receptor antagonist alleles in attention deficit hyperactivity disorder. *Mol Psychiatry*. **7,** 72-74 (2002).

134. Weissflog, L. *et al*. KCNIP4 as a candidate gene for personality disorders and adult ADHD. *Eur Neuropsychopharmacol*. **23,** 436-447 (2013).

135. Lanktree, M. *et al*. Association study of brain-derived neurotrophic factor (BDNF) and LIN-7 homolog (LIN-7) genes with adult attention-deficit/hyperactivity disorder. *Am J Med Genet B Neuropsychiatr Genet*. **147B,** 945-951 (2008).

136. Guimaraes, A. P. *et al*. MAOA is associated with methylphenidate improvement of oppositional symptoms in boys with attention deficit hyperactivity disorder. *Int J Neuropsychopharmacol*. **12,** 709-714 (2009).

137. Li, J. *et al*. The monoamine oxidase B gene exhibits significant association to ADHD. *Am J Med Genet B Neuropsychiatr Genet*. **147,** 370-374 (2008).

138. Domschke, K. *et al*. Association analysis of the monoamine oxidase A and B genes with attention deficit hyperactivity disorder (ADHD) in an Irish sample: preferential transmission of the MAO-A 941G allele to affected children. *Am J Med Genet B Neuropsychiatr Genet*. **134B,** 110-114 (2005).

139. Jiang, S. *et al*. Linkage studies between attention-deficit hyperactivity disorder and the monoamine oxidase genes. *Am J Med Genet*. **105,** 783-788 (2001).

140. Lowe, N., Hawi, Z., Fitzgerald, M. & Gill, M. No evidence of linkage or association between ADHD and DXS7 locus in Irish population. *Am J Med Genet*. **105,** 394-395 (2001).

141. Sánchez-Mora, C. *et al*. Evaluation of single nucleotide polymorphisms in the miR-183-96-182 cluster in adulthood attention-deficit and hyperactivity disorder (ADHD) and substance use disorders (SUDs). *Eur Neuropsychopharmacol*. **23,** 1463-1473 (2013).

142. Barr, C. L. *et al*. Linkage study of polymorphisms in the gene for myelin oligodendrocyte glycoprotein located on chromosome 6p and attention deficit hyperactivity disorder. *Am J Med Genet*. **105,** 250-254 (2001).

143. Ergul, E., Sazci, A. & Kara, I. Methylenetetrahydrofolate reductase gene polymorphisms in Turkish children with attention-deficit/hyperactivity disorder. *Genet Test Mol Biomarkers*. **16,** 67-69 (2012).

144. Hoogman, M. *et al*. Nitric oxide synthase genotype modulation of impulsivity and ventral striatal activity in adult ADHD patients and healthy comparison subjects. *Am J Psychiatry*. **168,** 1099-1106 (2011).

145. Kittel-Schneider, S. *et al*. Multi-level biomarker analysis of nitric oxide synthase isoforms in bipolar disorder and adult ADHD. *J Psychopharmacol*. **29,** 31-38 (2015).

146. Reif, A. *et al*. Influence of functional variant of neuronal nitric oxide synthase on impulsive behaviors in humans. *Arch Gen Psychiatry*. **66,** 41-50 (2009).

147. Okamura, N. *et al*. Gender-specific association of a functional coding polymorphism in the Neuropeptide S receptor gene with panic disorder but not with schizophrenia or attention-deficit/hyperactivity disorder. *Prog Neuropsychopharmacol Biol Psychiatry*. **31,** 1444-1448 (2007).

148. Fisher, S. E. *et al*. A genomewide scan for loci involved in attention-deficit/hyperactivity disorder. *Am J Hum Genet*. **70,** 1183-1196 (2002).

149. Lesch, K. P. *et al*. Genome-wide copy number variation analysis in attention-deficit/hyperactivity disorder: association with neuropeptide Y gene dosage in an extended pedigree. *Mol Psychiatry*. **16,** 491-503 (2011).

150. Kortmann, G. L. *et al*. The role of a mineralocorticoid receptor gene functional polymorphism in the symptom dimensions of persistent ADHD. *Eur Arch Psychiatry Clin Neurosci*. **263,** 181-188 (2013).

151. Smith, K. M., Bauer, L., Fischer, M., Barkley, R. & Navia, B. A. Identification and characterization of human NR4A2 polymorphisms in attention deficit hyperactivity disorder. *Am J Med Genet B Neuropsychiatr Genet*. **133B,** 57-63 (2005).

152. Cho, S. C. *et al*. Neurotrophin-3 gene, intelligence, and selective attention deficit in a Korean sample with attention-deficit/hyperactivity disorder. *Prog Neuropsychopharmacol Biol Psychiatry*. **34,** 1065-1069 (2010).

153. Park, S. *et al*. Neurotrophin 3 genotype and emotional adverse effects of osmotic-release oral system methylphenidate (OROS-MPH) in children with attention-deficit/hyperactivity disorder. *J Psychopharmacol*. **28**(3)**:** 220-226 (2014).

154. Pazvantoglu, O. *et al*. The relationship between the presence of ADHD and certain candidate gene polymorphisms in a Turkish sample. *Gene*. **528,** 320-327 (2013).

155. Carpentier, P. J. *et al*. Shared and unique genetic contributions to attention deficit/hyperactivity disorder and substance use disorders: a pilot study of six candidate genes. *Eur Neuropsychopharmacol*. **23,** 448-457 (2013).

156. Park, J. *et al*. Evidence that genetic variation in the oxytocin receptor (OXTR) gene influences social cognition in ADHD. *Prog Neuropsychopharmacol Biol Psychiatry*. **34,** 697-702 (2010).

157. Laurin, N. *et al*. No evidence for genetic association between DARPP-32 (PP1R1B) polymorphisms and attention deficit hyperactivity disorder. *Am J Med Genet B Neuropsychiatr Genet*. **147,** 339-342 (2008).

158. Jacob, C. *et al*. PPP2R2C as a candidate gene of a temperament and character trait-based endophenotype of ADHD. *Atten Defic Hyperact Disord*. **4,** 145-152 (2012).

159. Asherson, P. *et al*. A high-density SNP linkage scan with 142 combined subtype ADHD sib pairs identifies linkage regions on chromosomes 9 and 16. *Mol Psychiatry*. **13,** 514-521 (2008).

160. Brookes, K. J., Knight, J., Xu, X. & Asherson, P. DNA pooling analysis of ADHD and genes regulating vesicle release of neurotransmitters. *Am J Med Genet B Neuropsychiatr Genet*. **139B,** 33-37 (2005).

161. Turic, D. *et al*. A family based study implicates solute carrier family 1-member 3 (SLC1A3) gene in attention-deficit/hyperactivity disorder. *Biol Psychiatry*. **57,** 1461-1466 (2005).

162. Mick, E., McGough, J. J., Middleton, F. A., Neale, B. & Faraone, S. V. Genome-wide association study of blood pressure response to methylphenidate treatment of attention-deficit/hyperactivity disorder. *Prog Neuropsychopharmacol Biol Psychiatry*. **35,** 466-472 (2011).

163. English, B. A. *et al*. Choline transporter gene variation is associated with attention-deficit hyperactivity disorder. *J Neurodev Disord*. **1,** 252-263 (2009).

164. Kim, B. N. *et al*. Possible association of norepinephrine transporter -3081(A/T) polymorphism with methylphenidate response in attention deficit hyperactivity disorder. *Behav Brain Funct*. **6,** 57 (2010).

165. Kooij, J. S. *et al*. Response to methylphenidate in adults with ADHD is associated with a polymorphism in SLC6A3 (DAT1). *Am J Med Genet B Neuropsychiatr Genet*. **147B,** 201-208 (2008).

166. Lee, S. H. *et al*. Lack of association between response of OROS-methylphenidate and norepinephrine transporter (SLC6A2) polymorphism in Korean ADHD. *Psychiatry Res*. **186,** 338-344 (2011).

167. Park, S. *et al*. Possible effect of norepinephrine transporter polymorphisms on methylphenidate-induced changes in neuropsychological function in attention-deficit hyperactivity disorder. *Behav Brain Funct*. **8,** 22 (2012).

168. Yang, L., Wang, Y. F., Li, J. & Faraone, S. V. Association of norepinephrine transporter gene with methylphenidate response. *J Am Acad Child Adolesc Psychiatry*. **43,** 1154-1158 (2004).

169. Seeger, G., Schloss, P. & Schmidt, M. H. Marker gene polymorphisms in hyperkinetic disorder--predictors of clinical response to treatment with methylphenidate? *Neurosci Lett*. **313,** 45-48 (2001).

170. Thakur, G. A., Grizenko, N., Sengupta, S. M., Schmitz, N. & Joober, R. The 5-HTTLPR polymorphism of the serotonin transporter gene and short term behavioral response to methylphenidate in children with ADHD. *BMC Psychiatry*. **10,** 50 (2010).

171. McGough, J. *et al*. Pharmacogenetics of methylphenidate response in preschoolers with ADHD. *J Am Acad Child Adolesc Psychiatry*. **45,** 1314-1322 (2006).

172. Weber, H. *et al*. SPOCK3, a risk gene for adult ADHD and personality disorders. *Eur Arch Psychiatry Clin Neurosci*. **264,** 409-421 (2014).

173. Brookes, K. J. *et al*. Association of the steroid sulfatase (STS) gene with attention deficit hyperactivity disorder. *Am J Med Genet B Neuropsychiatr Genet*. **147B,** 1531-1535 (2008).

174. Brookes, K. J. *et al*. Polymorphisms of the steroid sulfatase (STS) gene are associated with attention deficit hyperactivity disorder and influence brain tissue mRNA expression. *Am J Med Genet B Neuropsychiatr Genet*. **153B,** 1417-1424 (2010).

175. Stergiakouli, E. *et al*. Steroid sulfatase is a potential modifier of cognition in attention deficit hyperactivity disorder. *Genes Brain Behav*. **10,** 334-344 (2011).

176. Kenar, A. N., Ay, O. I., Herken, H. & Erdal, M. E. Association of VAMP-2 and Syntaxin 1A Genes with Adult Attention Deficit Hyperactivity Disorder. *Psychiatry Investig*. **11,** 76-83 (2014).

177. Olgiati, P. *et al*. Role of synaptosome-related (SNARE) genes in adults with attention deficit hyperactivity disorder. *Psychiatry Res*. **215,** 799-800 (2014).

178. Kenar, A. N., Edgunlu, T., Herken, H. & Erdal, M. E. Association of synapsin III gene with adult attention deficit hyperactivity disorder. *DNA Cell Biol*. **32,** 430-434 (2013).

179. Makkar, R. *et al*. The gene for synapsin III and attention-deficit hyperactivity disorder. *Psychiatr Genet*. **17,** 109-112 (2007).

180. Liu, L. *et al*. Association between SYP with attention-deficit/hyperactivity disorder in Chinese Han subjects: differences among subtypes and genders. *Psychiatry Res*. **210,** 308-314 (2013).

181. Vanti, W. B. *et al*. Discovery of a null mutation in a human trace amine receptor gene. *Genomics*. **82,** 531-536 (2003).

182. Yan, T. C. *et al*. NK1 (TACR1) receptor gene 'knockout' mouse phenotype predicts genetic association with ADHD. *J Psychopharmacol*. **24,** 27-38 (2010).

183. Comings, D. E. *et al*. Exon and intron variants in the human tryptophan 2,3-dioxygenase gene: potential association with Tourette syndrome, substance abuse and other disorders. *Pharmacogenetics*. **6,** 307-318 (1996).

184. Comings, D. E., Gade, R., Muhleman, D. & Sverd, J. No association of a tyrosine hydroxylase gene tetranucleotide repeat polymorphism in autism, Tourette syndrome, or ADHD. *Biol Psychiatry*. **37,** 484-486 (1995).

185. Barr, C. L. *et al*. Further evidence from haplotype analysis for linkage of the dopamine D4 receptor gene and attention-deficit hyperactivity disorder. *Am J Med Genet*. **96,** 262-267 (2000).

186. Johansson, S. *et al*. Common variants in the TPH1 and TPH2 regions are not associated with persistent ADHD in a combined sample of 1,636 adult cases and 1,923 controls from four European populations. *Am J Med Genet B Neuropsychiatr Genet*. **153B,** 1008-1015 (2010).

187. Tang, G. *et al*. Lack of association between the tryptophan hydroxylase gene A218C polymorphism and attention-deficit hyperactivity disorder in Chinese Han population. *Am J Med Genet*. **105,** 485-488 (2001).

188. Li, J. *et al*. Association between tryptophan hydroxylase gene polymorphisms and attention deficit hyperactivity disorder in Chinese Han population. *Am J Med Genet B Neuropsychiatr Genet*. **141B,** 126-129 (2006).

189. Manor, I. *et al*. Association between tryptophan hydroxylase 2, performance on a continuance performance test and response to methylphenidate in ADHD participants. *Am J Med Genet B Neuropsychiatr Genet*. **147B,** 1501-1508 (2008).

190. Xu, X *et al*. Investigation of the ZNF804A gene polymorphism with genetic risk for bipolar disorder in attention deficit hyperactivity disorder. *BMC Res Notes*. **6,** 29 (2013)
